# One Blob to Rule Them All: A Universal Dual-Topology Nonequilibrium Framework for Relative and Absolute Binding Free Energies via a Lennard-Jones Blob Reference

**DOI:** 10.1021/acs.jctc.6c00932

**Published:** 2026-08-12

**Authors:** Piero Procacci

**Affiliations:** Dipartimento di Chimica “Ugo Schiff”, Università degli Studi di Firenze, Via della Lastruccia 3, 50019 Sesto Fiorentino, Italy

## Abstract

Single-topology (ST) free energy perturbation (FEP) methods, while accurate for congeneric series, require chemical similarity between ligands, bespoke perturbation networks, and offer no direct route to absolute binding free energies (ABFEs). Here we introduce DT-NE-Alchemy, a dual-topology nonequilibrium (NE) framework that eliminates these constraints through a universal Lennard-Jones (LJ) blob reference state. The LJ blob—a minimal, rigid molecule (as small as a single LJ particle) designed to fit the binding pocket—serves as a common anchor for alchemical transformations. Relative binding free energies between any two ligands are obtained by two-edge transformations *L*_*i*_ → blob → *L*_*j*_, yielding all *n*(*n* – 1)/2 pairwise values from only *n* blob-based calculations. Crucially, ABFEs follow directly as $\Delta G_{blob}+\Delta G_{blob\to L_{i}}$ , converting the long-sought “holy grail” of computational drug design into a practical reality. Applied to 16 MCL-1 ligands spanning indole, benzothiophene, and benzofuran scaffolds, the method achieves Pearson correlations up to 0.88, Kendall τ of ≃0.70, and mean unsigned errors of 1.2–1.5 kcal/mol for 120 relative pairs. The methodology delivers credible, tunable confidence intervals directly from BAR analysis, controllable via switching time τ and trajectory count *N* without costly replicate simulations. All computational tasks are embarrassingly parallel by design, perfectly aligned with leadership-class HPC systems. DT-NE-Alchemy is automatable and poised to effectively complement ST/FEP for the exploration of chemically diverse libraries and scaffold identification, with the latter remaining the gold standard for lead optimization within strictly congeneric series.

## Graphical abstract

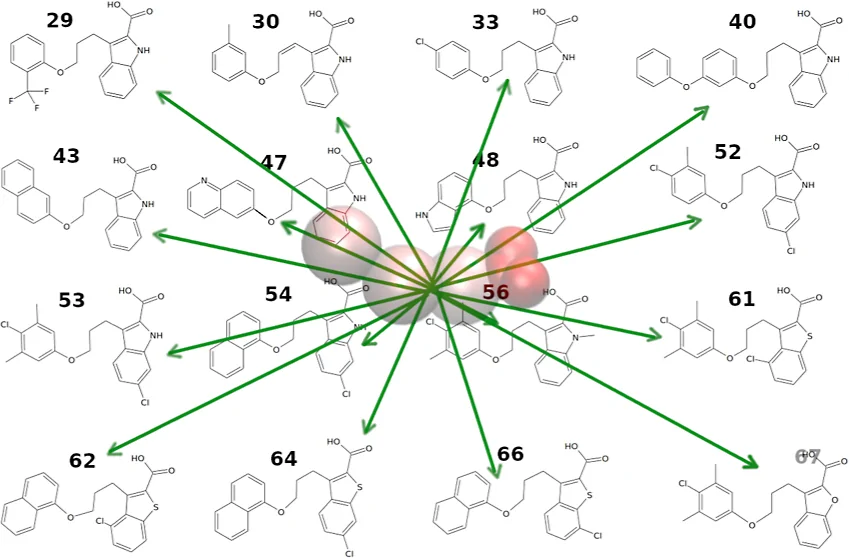

## 1 Introduction

In the past decade, the steady rise of computer power, boosted by the advent of graphical processing units (GPUs) in heterogeneous high-performance computing (HPC) facilities, and the constant improvement in efficiency, accuracy, and reliability of algorithms and protocols^1–5^ have made in silico screening competitive with traditional medicinal chemistry practice in early preclinical stages.^6,7^ In this context, alchemical free energy methods based on atomistic molecular dynamics simulations^8–13^ using free energy perturbation (FEP) are today considered the gold standard for binding free energy predictions, often serving as the final confirmatory step for lead identification in the in silico drug discovery pipeline.

The primary advantage of alchemical methodologies lies in their ability to replace the physically complex process of ligand–protein binding with a series of computationally tractable, albeit nonphysical (“alchemical”), transformations. By decoupling the ligand from its environment through a thermodynamic cycle, these methods rigorously account for the entropic and enthalpic contributions that govern molecular recognition. In principle, the alchemical approach can evaluate the absolute binding free energy (ABFE) as the difference between the solvation free energy of the ligand in the bound state and in bulk. As schematically depicted in Figure 1a, these solvation free energies are computed by progressively decoupling the ligand from the environment via a λ parameter that regulates the ligand-environment interaction potential, using a stratification^8^ of discrete *equilibrium* intermediate λ-states with FEP^14^ or, equivalently, thermodynamic integration^15^ (TI).

**Figure 1:**
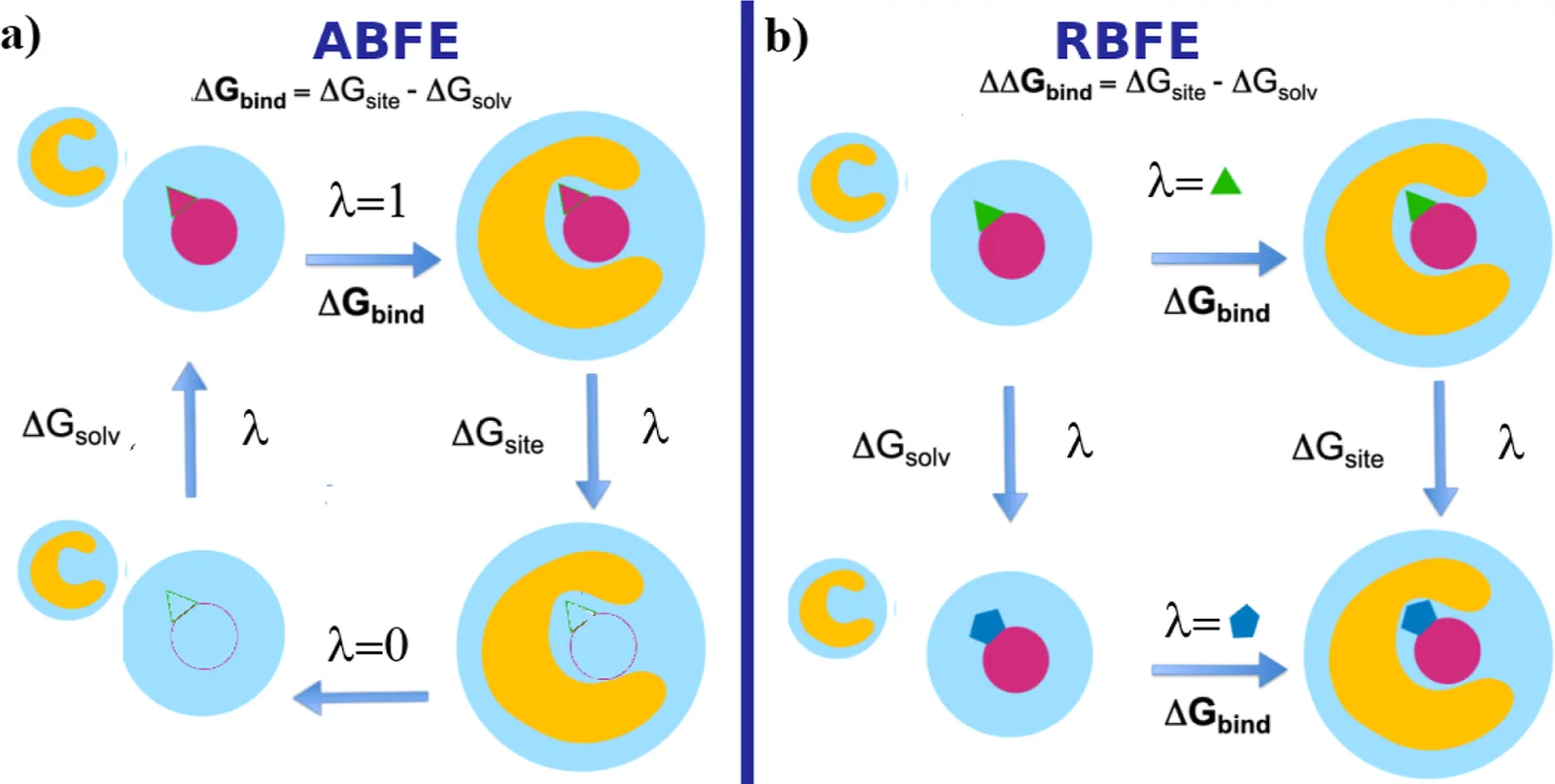
Thermodynamic cycles for (a) ABFE and (b) RBFE calculations.

While the statistical mechanical foundation of alchemically computed binding affinities is rigorous,^16–18^ the practical calculation of ABFEs using standard alchemical techniques still faces daunting challenges. FEP calculations are prohibitively expensive and struggle to achieve the accuracy required (consistently within 1 kcal/mol) for reliable data generation at scale. ABFE calculations using FEP remain fundamentally unreliable—with errors of approximately 2–3 kcal/mol at best—even for relatively simple systems such as host–guest pairs (see, e.g., the results of the *blind* SAMPL challenges^19–22^ over the past decade). This unreliability stems from the large thermodynamic perturbations involved (tens of kcal/mol in both legs of the alchemical cycle), the challenges in achieving true Boltzmann sampling along the alchemical stratification, and the difficulties in handling corrections due to the standard state volume^16,18,23^ and net charge changes.^24–26^

In industrial settings, MD-based alchemical methods are generally used for ranking the *relative* binding affinities of a series of *strictly congeneric compounds* to a protein target, in order to prioritize them for further wet-lab assessment.^27,28^ Relative binding free energy (RBFE) calculations are also performed using FEP via a thermodynamic cycle (see Figure 1b) where compound A is transmuted into a parent compound B in the bound state and in pure solvent. These alchemical transformations require a common core and a predefined perturbation map, with changes involving a few small substituents. The thermodynamic perturbation is in this case at most a few kcal/mol in both legs of the cycle, dramatically improving the precision and reproducibility of the calculation. These features make RBFE inherently more accurate than ABFE, becoming, de facto, the workhorse in industrial lead optimization.^7,29^ However, the limitations stemming from the strict applicability of alchemical RBFE calculations to congeneric series are de facto akin to those of medicinal chemistry itself, where a common core scaffold and late-stage diversification are prerequisites for synthetic practicability.

The current consensus in RBFE calculations is based on the so-called single-topology (ST) scheme, whereby the two compounds share a common topological structure with the same number of atoms, and a single noncore atom is transformed into another (e.g., H into a halogen atom). When the number of atoms differs, as in an R-group perturbation^30,31^ (e.g., H into CH_3_ or CH into N in an aromatic ring), “dummy” atoms are added to the common core structure. While these atoms are fully interacting in one end-state, in the other end-state they are bound to the molecule with zero nonbonded parameters in the bound and unbound states, although they are still coupled to the real atoms via the bonded potential. The effect of the dummy atoms should not influence the RBFE provided that this spurious contribution cancels in the two arms of the thermodynamic cycle.^30^

To alleviate the phase space mismatch and thermodynamic instability typical of scaffold hopping in relative binding free energy (RBFE) calculations, several FEP-based methodologies have emerged within alchemical frameworks, including hybrid single-dual topology schemes,^32^ Alchemical transfer model (ATM),^33^ QligFEP workflow,^34^ and the separated topologies approach.^35^ In most of these dual-topology schemes, the two ligands are both simultaneously present in the complex, each retaining its own topology and coordinates. In one end-state one ligand is fully coupled while the other is completely decoupled (“ghost” state). During the alchemical transformation the former is progressively decoupled while the latter is recoupled. Structural collapse of disappearing atoms is avoided using either soft-core potentials^32,34–36^ or, in the ATM framework, by introducing a coordinate displacement vector that swaps the positions of the bound and unbound ligands without requiring soft-core regularization.^33^

In two recent studies^37,38^ we proposed a novel dual-topology (DT) scheme for RBFE calculations between dissimilar molecules. RBFEs are efficiently achieved using bidirectional nonequilibrium (NE) DT schemes, which handle disparate topologies, net charges, and volumes. This bidirectional approach enhances sampling via Hamiltonian replica exchange (HREM) at the end-states and uses fast nonequilibrium transitions to compute work distributions, applying the Crooks fluctuation theorem^39^ and the Bennett acceptance ratio^40^ to recover the RBFEs. The method was applied to predict the RBFEs of the host–guest systems recently proposed in the SAMPL9 challenge,^41^ yielding a Pearson correlation coefficient of ρ ≃ 0.9 and a mean unsigned deviation (MUD) of ≃1.5 kcal/mol. These results were obtained for RBFEs involving pairs with Tanimoto indices ranging from 0.17 to 0.80, transforming small saturated amines into polycyclic aromatic or bulky cage compounds such as adamantyl derivatives, thereby bypassing completely the limitations of current ST-FEP technologies for congeneric series.

In this paper, we further elaborate on the DT-NE technology by introducing a receptor-specific universal alchemical reference—an approriately designed “LJ blob”. Conceptually, this strategy is reminiscent of the one-step perturbation (OSP) reference-state methodology,^42,43^ which also employed an artificial reference state to connect structurally diverse ligands.^42,43^

Using this common thermodynamic reference, RBFEs between any two ligands (say, A and B) can be accessed via two-edge transmutations of the kind A → LJ blob → B. The minimalistic topology and shape of the LJ blobs can be designed to fit efficiently into the cavity receptor under scrutiny. The chemical simplicity and reduced size of these receptor-specific reference compounds result in reduced dissipation during the DT-NE forward and reverse NE processes, affording precise and reproducible RBFEs with credible confidence intervals. An additional advantage of the blob-based strategy is that the one-edge DT-NE calculations immediately provide the ligand ABFEs *up to an additive constant*, namely the ABFE of the reference blob itself. The latter can be computed using efficient nonequilibrium techniques such as virtual double system single box (vDSSB),^44–47^ a calculation that, for suitably designed blobs, is considerably less demanding than computing the ABFE of a flexible drug-like ligand.

The blob-based DT-NE methodology has been applied to calculate all the RBFEs among 16 ligands of the MCL-1 protein^4^ using several reference LJ-blobs and two general force field for a total of more than 150 independent DT-NE calculation (see also data reported in the Zenodo repository https://zenodo.org/records/21418117). The 16 MCL-1 ligands are strictly congeneric and share a bulky common scaffold. They are therefore ideally suited for optimized perturbation networks using conventional ST-FEP. For this very reason, the MCL-1 benchmark constitutes a *stringent stress test* for the blob-based DT-NE methodology. The similarity of the ligands is a property of the benchmark, not an assumption of the DT-NE approach, since every calculation transforms a ligand into an artificial blob whose Tanimoto similarity with the ligands is systematically close to zero. Results obtained for the RBFEs and ABFEs of the MCL-1 series,^48^ are extremely encouraging, with Pearson correlation coefficients consistently close to 0.8, Kendall ranking beyond 0.6 and MUDs of the order of 1 kcal/mol, showing a remarkable independence on the selected reference blob for the DT-NE calculations.

The methodology presented herein represents, in our view, a significant step toward extending rigorous alchemical free-energy calculations beyond the congeneric regime traditionally accessible to single-topology approaches. By reducing the problem to the precise determination of the ABFE of a minimal, well-behaved reference compound—the LJ blob—and leveraging the inherent parallelism of HREM sampling and NE transformations, our approach circumvents the convergence bottlenecks and charge artifacts that have historically plagued alchemical ABFE calculations. Furthermore, the universal nature of the LJ blob reference for intermediate RBFEs eliminates the need for chemical similarity constraints or bespoke perturbation networks, rendering the entire workflow eminently automatable. For a given protein target, the optimal blob geometry can be derived directly from the binding pocket structure (e.g., by identifying a minimal molecular fragment that occupies the deepest subpockets), enabling a fully automated, high-throughput pipeline. Once the reference state is defined, ABFEs for any ligand library can be generated at scale on HPC architectures with minimal human intervention, with each ligand processed independently in an embarrassingly parallel fashion. The synergy between blob-based DT-NE alchemy for scaffold exploration^37^ and conventional ST-NE alchemy^49^ or ST-FEP+^4^ for lead optimization within congeneric series may provide a practical and physically grounded workflow for next-generation structure-based drug discovery.

The paper is organized as follows. In the “Theory” section, we provide the thermodynamic foundation of the DT-NE technology. Full methodological details are provided in the Supporting Information. In the section “Results” we present our findings for the RBFEs of the MCL-1 ligands using various LJ blob designs and parametrizing the ligands with both the AM1-BCC model^50^ and the successor ABCG2 force field. In particular, in Subsections RBFE with the blob reference and Cycle closure conditions, the thermodynamic consistency of the methodology is assessed, while in the following subsections DT-NE RBFEs and ABFEs using are compared to the experimental data.

In the Discussion section, we place the blob-based DT-NE methodology in the context of recent dual-topology and reference-state approaches. Similarities, differences, strengths, and limitations of the various approaches are explicitly discussed. Conclusive remarks and future directions are provided in the Conclusion Section.

## 2 Theory

### 2.1 RBFE via NE Alchemical Simulations

In NE alchemical simulations, RBFEs are achieved by performing two sequential steps: (i) Boltzmann sampling of the end-states via HREM solute tempering, followed by (ii) generation of a swarm of, say *N*, independent NE alchemical trajectories—initiated from the configurations collected in step (i)—where compound A is gradually transformed into compound B, and vice versa, in both arms of the thermodynamic cycle (see Figure 1). The alchemical transformation in each NE trajectory is driven using a common time schedule via a λ-coupling parameter that regulates the interaction potential of the transmuting ligand with the environment. The NE swarms—forward (A → B) and reverse (B → A)—produce the work distributions *P*_A→B_(*W*|*F*) and *P*_B→A_(*W*|*R*). The difference between the mean work ⟨*W*_A→B_⟩ (or ⟨*W*_B→A_⟩) and the corresponding underlying free energy Δ*G*_A→B_ (or Δ*G*_B→A_) is called *dissipation*. While some NE trajectories may yield a work *W* smaller than Δ*G*, the dissipation *W*_d_ = ⟨*W*⟩ – Δ*G* is a definite positive quantity, in accordance with the second law of thermodynamics. For a Markovian process, the inverse of the dissipation grows *linearly* with the duration τ of the NE process (i.e., the switching time):^51^ the shorter the time τ, the larger the dissipation.

The relative binding free energy of the *forward* process Δ*G*_A→B_—i.e., the free energy to transform A into B—can be recovered from the collection of work values using the Bennett acceptance ratio (BAR)^40,52^ by solving the equation

1 $$\sum_{i=1}^{N}\frac{1}{1+e^{\beta \left(W_{i}\left(A\to B\right)-\Delta G_{A\to B}\right)}}-\sum_{i=1}^{N}\frac{1}{1+e^{-\beta \left(W_{i}\left(B\to A\right)+\Delta G_{A\to B}\right)}}=0$$

where β = 1/*k*_B_ *T* is the inverse Boltzmann temperature. Equation 1 is valid provided that the time-dependent coupling protocol λ­(*t*) for the forward transformation A → B and its time-reversed counterpart λ­(τ – *t*) for B → A are strictly mirrored. The error in the calculation of Δ*G*_A→B_ via BAR can be reliably estimated either by bootstrapping with resampling or by using the analytical formula for the variance proposed by Shirts et al. (see eq 10 in ref 52). The BAR formula can be derived from the Crooks fluctuation theorem^39^

2 $$\frac{P_{A\to B}\left(W|F\right)}{P_{B\to A}\left(-W|F\right)}=e^{\beta \left(W_{A\to B}-\Delta G_{A\to B}\right)}$$

using a maximum-likelihood argument.^52^ eq 2 states that the RBFE Δ*G*_A→B_ corresponds to the *crossing point* of the forward work distribution and the mirror-symmetric reverse work distribution. For finite work samples, the precision of the BAR estimate of this crossing, eq 1, depends on the extent of the overlap between the two distributions, which decreases with increasing total dissipation *W*_d_ = *W*_d_(A → B) + *W*_d_(B → A). For overlapping distributions, the confidence interval of the BAR estimate decreases with the number of work values as *N* ^–1/2^, and no other estimator (e.g., the Jarzynski equality^53^) can produce a Δ*G*_A→B_ with a smaller statistical error. When the work distributions *P*_A→B_(*W*|*F*) and *P*_B→A_(−*W*|*F*) have little or no overlap, the BAR estimate becomes essentially equivalent to the mean of the forward and reverse Jarzynski estimates, which are both biased and crucially affected by the left and right tails of the respective histograms.^54^

It is important to stress that, while the bidirectional BAR estimate is *unbiased* in the case of overlapping distributions, its accuracy depends *critically* on the quality of sampling of the end-states. Sampling issues are known to plague methodologies such as FEP,^55–59^ which are based on *equilibrium* MD simulations over a stratification of a finite number of intermediate λ states. In NE-Alchemy, the sampling challenge persists, but *it is now confined solely to the end-states*—specifically, to the initial configurations from which the NE trajectories are launched. This initial equilibrium sampling is the only sampling that matters in NE alchemy. Once the nonequilibrium transitions begin, sampling is no longer an issue: we leave the equilibrium FEP paradigm and enter a new world governed by the Jarzynski and Crooks theorems. The trajectories terminate in states that are not canonically distributed. What is “sampled” during the alchemical switch is irrelevant. The final work distribution is the nonequilibrium fingerprint of the initial equilibrium sampling and of the dissipation inherent to the NE process.

### 2.2 Dual-Topology Approach in NE Alchemy Using the LJ-Blob as a Universal Reference for RBFE Determination

In industrial and academic settings, most production pipelines for RBFE calculations target *strictly congeneric compounds* using free energy perturbation (FEP) within the single-topology (ST) framework. In the most advanced implementations, sampling along the λ stratification in the bound state is enhanced via Hamiltonian replica exchange with a solute tempering method involving a “hot region” that encompasses the ligand and the binding pocket (FEP+).^4^ While the FEP+-ST approach can deliver accurate and reproducible RBFEs (aside from systematic force field errors) thanks to the small thermodynamic perturbations involved, the ST scheme imposes a strict requirement of chemical similarity between the two compounds. This limitation severely restricts the reach of the methodology for data generation at scale in drug discovery screening campaigns.

Recently, in refs 37 and 38 we tested a dual-topology (DT) alchemical scheme for host–guest systems. In this DT approach, both ligands are present simultaneously during the NE alchemical transitions: one ligand gradually decouples from the environment while the other recouples. In the end-states, the conformation and orientation of the coupled ligand are determined by the environment (binding site or pure solvent), whereas the “ghost” (decoupled) ligand is sampled from a simulation of the *isolated* molecule. To reduce energetic stress during the bound-state NE transitions, *the two ligands do not interact with each other* but only with the environment. This allows them to “pass through” one another, a feature essential for handling noncongeneric (chemically distant) compounds. This also allows the initially decoupled ligand to grow freely in the binding site, which is kept devoid of water or protein residues by the initially coupled ligand.

Crucially, in the bound-state end-states, both the ghost and the interacting ligand are held in the binding site by a harmonic tethering potential that connects *their centers of mass* (COM)

3 $$U_{AB}=\frac{1}{2}K{\left|r_{A}^{COM}-r_{B}^{COM}\right|}^{2}$$

The tethering energy vanishes when the two COMs coincide. De facto, eq 3 is a restraint that keeps both ligands in the binding site *without involving the protein atoms*. Also, because internal molecular motions are orthogonal to the COM translation, the conformational behavior of the coupled ligand is unaffected by the harmonically bound ghost ligand, and vice versa. As rigorously demonstrated in ref 37 the bound-state RBFE is independent of the restraint’s presence. However, the restraint may affect *dissipation* during the alchemical transformation, especially if the equilibrium poses of the two ligands in the binding site are not coincident. In that case, the recoupling ghost ligand must drag the decoupling partner toward its actual position, dissipating energy along the alchemical path. It is important to stress that such path-dependent side effects have no consequence on the free energy, which is a state function of the end-states only.

Dissipation during the decoupling of one ligand and the recoupling of the partner is minimized by two key features: (i) the two ligands do not interact with each other during the transition, and (ii) a modified soft-core regularization^36^ based on *shifted*, rather than softened, electrostatic and Lennard-Jones potentials

4 $$\begin{array}{l} v_{LJ}\left(r\right)=4\epsilon \left(1-\lambda \right)\left[{\left(\frac{\sigma }{r+\lambda \alpha }\right)}^{12}-{\left(\frac{\sigma }{r+\lambda \alpha }\right)}^{6}\right]\\ v_{el}\left(r\right)=\left(1-\lambda \right)\frac{q_{i}q_{j}}{4\pi \epsilon_{0}\left(r+\lambda \beta \right)} \end{array}$$

with shift parameters α = 0.35 Å and β = 4.0 Å tuned to minimize dissipation.

Within this DT-NE framework, one can devise an alchemical technology that employs a *universal reference ligand*—the **LJ blob**—chosen to be as simple and tame as possible from structural and conformational viewpoints to fit the binding site. This universal reference can then be used to probe any set of ligands, *no matter how dissimilar they are*.

As illustrated in Figure 2b, the RBFE between *any* two real ligands *L*_*i*_ and *L*_*j*_ in the set can be straightforwardly recovered via a two-edge transformation

5 $$\begin{array}{l} \Delta G_{L_{i}\to L_{j}}=\Delta \Delta G_{L_{i}\to blob}+\Delta \Delta G_{blob\to L_{j}}\\ =\left(\Delta \Delta G_{L_{i}\to blob}^{b}-\Delta \Delta G_{L_{i}\to blob}^{u}\right)+\left(\Delta \Delta G_{blob\to L_{j}}^{b}-\Delta \Delta G_{blob\to L_{j}}^{u}\right) \end{array}$$

where all free energies are estimated via BAR, and $\Delta \Delta G_{L_{i}\to blob}^{b}$ and $\Delta \Delta G_{L_{i}\to blob}^{u}$ denote the free energy changes for transforming ligand *L*_*i*_ into the LJ blob in the bound state and in bulk solvent, respectively. Most importantly, eq 5 implies that the net charge of the reference LJ blob may differ from that of the real ligands: the two-edge calculation makes the blob’s charge irrelevant in eq 5. Thus, the blob’s charge, along with its LJ parameters and atomic partial charges, can be optimally tuned to fit the binding site and yield a physically sensible absolute binding free energy (*vide infra*).

**Figure 2:**
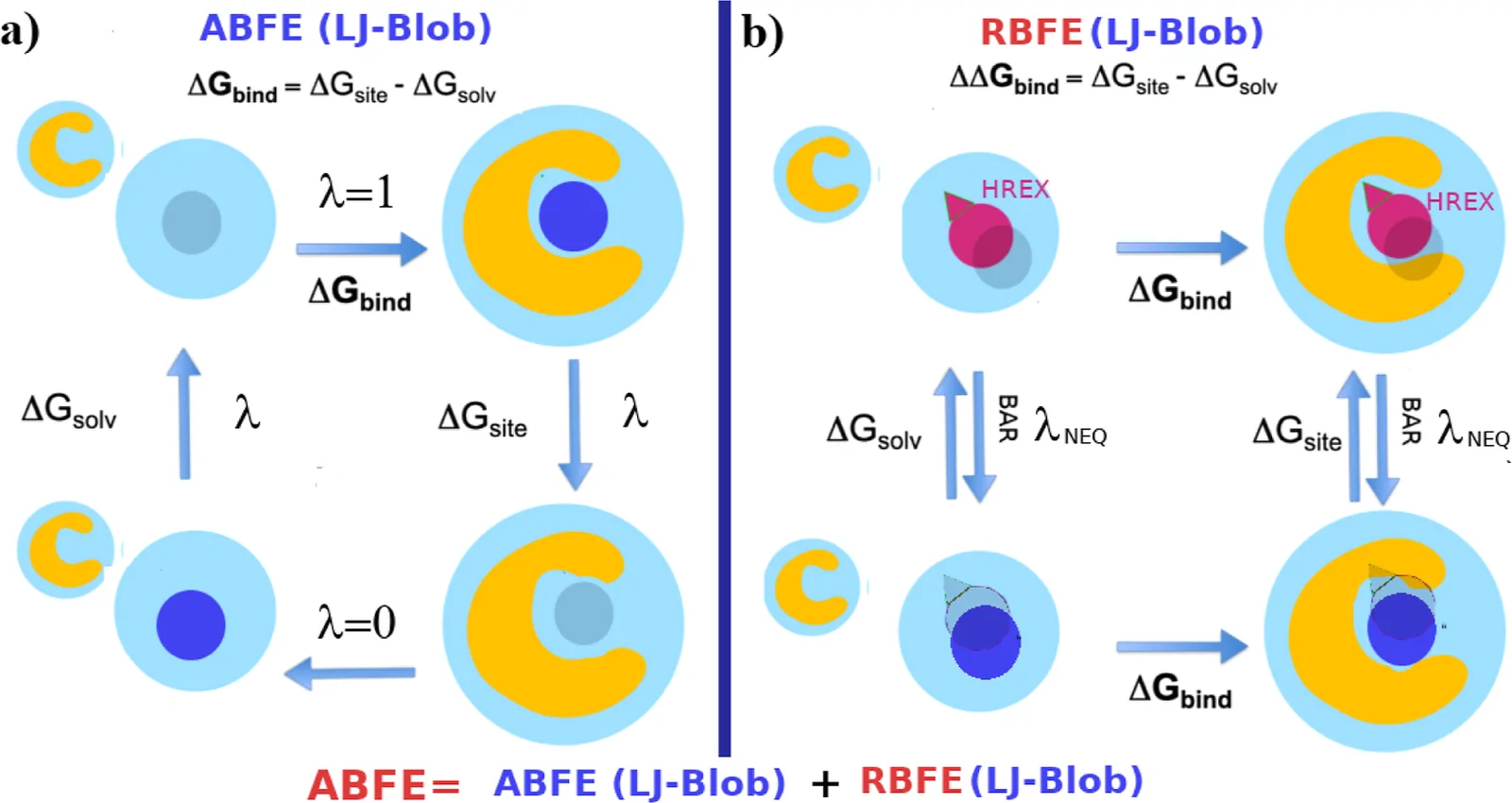
Thermodynamic cycles for (a) ABFE and (b) RBFE calculations.

The confidence interval for the estimate in eq 5 is obtained by summing in quadrature the errors of the four individual transformations. By appropriately choosing the switching time τ and the number *N* of work values, the variance of the BAR crossing point can be kept within approximately 1 kcal/mol. The uncertainty of the BAR estimate can be further reduced by combining the work values of the bound and unbound state to form the *N* ^2^ vDSSB convolution

$$\begin{array}{l} P^{vDSSB}{\left(W|F\right)}_{blob\to L}=P_{blob\to L}^{\left(b\right)}\times P_{L\to blob}^{\left(u\right)}\left(W\right)\\ P^{vDSSB}{\left(W|R\right)}_{L\to blob}=P_{L\to blob}^{\left(b\right)}\times P_{blob\to L}^{\left(u\right)}\left(W\right) \end{array}$$ 6

where the suffixes (b) and (u) refer to the bound and ubound states, so that

7 $$\Delta \Delta G_{L_{i}\to L_{j}}=\Delta \Delta G_{L_{i}\to blob}^{vDSSB}+\Delta \Delta G_{blob\to L_{j}}^{vDSSB}$$

where the blob → *L*_*i*_ RBFEs are computed using the convolutions eq 6. The confidence interval is in this case obtained by bootstrapping with resampling on the bound and unbound work samples *before* performing the convolution. As we will show in the Results section, the total error is largely dominated by the bound-state contributions $\Delta \Delta G_{blob\to L_{i}}^{b}$ .

For a set of *n* ligands, eq 5 (or eq 7) provides access to all *n*(*n* – 1)/2 possible RBFEs. This enables systematic error detection: systematic force field biases become apparent as outliers when comparing computed versus experimental RBFEs across the full set, as quantified by metrics such as the mean unsigned deviation.

### 2.3 From RBFE to Absolute Binding Free Energy via LJ Blob

Once the blob-to-ligand RBFEs $\Delta G_{blob\to L_{i}}$ have been determined, the absolute binding free energy of each ligand can be obtained directly

8 $$\Delta G_{L_{i}}^{0}=\Delta G_{blob}^{0}+\Delta \Delta G_{blob\to L_{i}}$$

where Δ*G*_blob_^0^ is the absolute binding free energy of the LJ blob itself, computed once using standard equilibrium techniques (e.g., FEP+) or nonequilibrium methods such as vDSSB. Critically, $\Delta \Delta G_{blob\to L_{i}}$ is not an arbitrary alchemical free energy difference but rather the difference between two absolute binding free energies, i.e. $\Delta \Delta G_{blob\to L_{i}}=\Delta G_{L_{i}}^{0}-\Delta G_{blob}^{0}$ . This relation follows directly from the thermodynamic cycle in Figure 2. Consequently, any blob force field nuances affecting Δ*G*_blob_^0^ cancel exactly in the determination of $\Delta \Delta G_{blob\to L_{i}}$ , because they appear in both $\Delta \Delta G_{blob\to L_{i}}$ and Δ*G*_blob_^0^ with opposite signs. The only place where the blob’s force field description enters the final ABFE is through the explicit term Δ*G*_blob_^0^ in eq 8. This term is computed once, and its associated error is primarily methodological—e.g., convergence of the alchemical protocol, handling of volume and charge corrections of the *ligand* when applicable (and not of the blob for which these corrections cancel out exactly, again by virtue of eq 8. Thus, Δ*G*_blob_^0^ *adds as a constant offset* to all ligands, yielding a ρ and Kendall rank coefficient τ for the ABFEs which are identical to those computed for the ordered set of the *n*(*n* – 1) real $\Delta \Delta G_{L_{i}\to L_{j}}$ with *i* ≠ *j*. As long as the blob lingers in the binding pocket in the bound state, the force field parameters of the blob itself are therefore largely irrelevant, as any inaccuracies they might introduce cancel out in the RBFE leg $\Delta \Delta G_{blob\to L_{i}}$ .

This cancellation represents a key advantage of the LJ-blob reference state: the reference compound does not need to be accurately described by the force field; it need only be small, conformationally rigid, and geometrically compatible with the binding site—i.e. a coarse-grained pharmacophore—to minimize methodological errors in the determination of Δ*G*_blob_^0^. The accuracy of the final ABFE thus depends primarily on (i) the precision of the NE-BAR estimates for the $\Delta \Delta G_{blob\to L_{i}}$ transformations and (ii) the methodological accuracy of the standalone Δ*G*_blob_^0^ calculation, with the latter contributing only a constant offset that does not affect relative ranking.

### 2.4 Cycle Closure Condition Using Pairs of LJ-Blobs

In alchemical free energy calculations, thermodynamic consistency requires that the free energy differences along any closed cycle sum to zero. For a set of ligands and a universal reference state, this cycle closure condition (CCC) provides a powerful internal check of the methodology’s accuracy and precision, independent of experimental data. Specifically, for any ligand *L* and two distinct LJ blobs (blob1 and blob2), thermodynamic consistency demands

9 $$\Delta \Delta G_{blob1\to L}+\Delta \Delta G_{L\to blob2}+\Delta \Delta G_{blob2\to blob1}=0$$

where each term is a free energy difference obtained from bidirectional NE simulations and BAR estimation.

To reliably assess the closure condition, we thus introduce a *second* LJ blob (blob2) with a different molecular composition or charge distribution compared to the original reference (blob1). Critically, evaluating eq 9 does *not* require resampling the end-states of the real ligands in the bound state, a computationally expensive step involving Hamiltonian replica exchange (HREM) simulations. Only the end-state sampling of the two blobs in the bound state and in bulk solvent is required. Since both blobs are small, conformationally rigid molecules, their equilibrium sampling is computationally inexpensive and converges rapidly. The NE alchemical transformations must, of course, be rerun for the new blob, but the computational overhead is modest given the blobs’ simplicity.

For a set of *n* ligands, eq 9 must hold for each ligand individually. We can therefore define the cycle closure residual for ligand *L* as

10 $$\epsilon \left(L\right)=\Delta \Delta G_{blob1\to L}+\Delta \Delta G_{L\to blob2}+\Delta \Delta G_{blob2\to blob1}$$

where ϵ­(*L*) is expected to be zero within statistical uncertainty if the methodology is thermodynamically consistent and free of systematic errors.

A useful consequence of the CCC is the relationship between the blob-to-ligand free energies obtained with the two different reference states. From eq 9, we have

11 $$\Delta \Delta G_{blob2\to L}-\Delta \Delta G_{blob1\to L}=\Delta \Delta G_{blob2\to blob1}=\Delta G_{blob2}^{0}-\Delta G_{blob1}^{0}$$

where Δ*G*_blob2_^0^ and Δ*G*_blob1_^0^ are the absolute binding free energies of the two blobs. Thus, the mean unsigned error (MUD) between ΔΔ*G*_blob1→*L*_ and ΔΔ*G*_blob2→*L*_ across the ligand set should equal the absolute difference $\left|\Delta G_{blob2}^{0}-\Delta G_{blob1}^{0}\right|$ , providing an independent cross-validation of the consistency of the method.

Most importantly, the average of the cycle closure residuals across all ligands

12 $$\left\langle \epsilon \right\rangle =\frac{1}{n}\sum_{i=1}^{n}\epsilon \left(L_{i}\right)$$

quantifies the overall thermodynamic consistency of the methodology. Unlike the “hysteresis” often reported in single-topology FEP calculations—which reflects a failure to achieve equilibrium at intermediate λ states—⟨ϵ⟩ in our framework provides a direct measure of the combined statistical and systematic errors inherent in the NE-BAR protocol. A nonzero ⟨ϵ⟩ indicates either insufficient convergence of the end-state sampling, inadequate dissipation reduction (i.e., too short switching times τ), or force field inconsistencies. For a well-converged calculation, we expect |⟨ϵ⟩| to be well within the typical BAR uncertainties of the individual two-edge transformations $\Delta \Delta G_{L_{i}\to L_{j}}=\Delta \Delta G_{L_{i}\to blob1}+\Delta \Delta G_{blob1\to L_{j}}$ . As we will demonstrate in the Results section, our protocol achieves cycle closure residuals that are statistically indistinguishable from zero, confirming the internal consistency of the NE-Alchemy approach.

## 3 Results

### 3.1 The System

We applied the DT-NE approach for RBFE and ABFE calculations via LJ blobs to 16 ligands of the MCL-1 protein with known dissociation constants,^60^ spanning the indole (ligands 29–56), benzothiophene (61–64), and benzofuran (67) families (see Figure 3). These ligands were also part of the “jacs-set” benchmark for RBFE calculations using equilibrium FEP+^4^ and, most recently, nonequilibrium ST-BAR-based^49^ methodologies. We stress once more that the similarity of the MCL-1 ligands is a property of the benchmark, not an assumption of the DT-NE/LJ-blob methodology.

**Figure 3:**
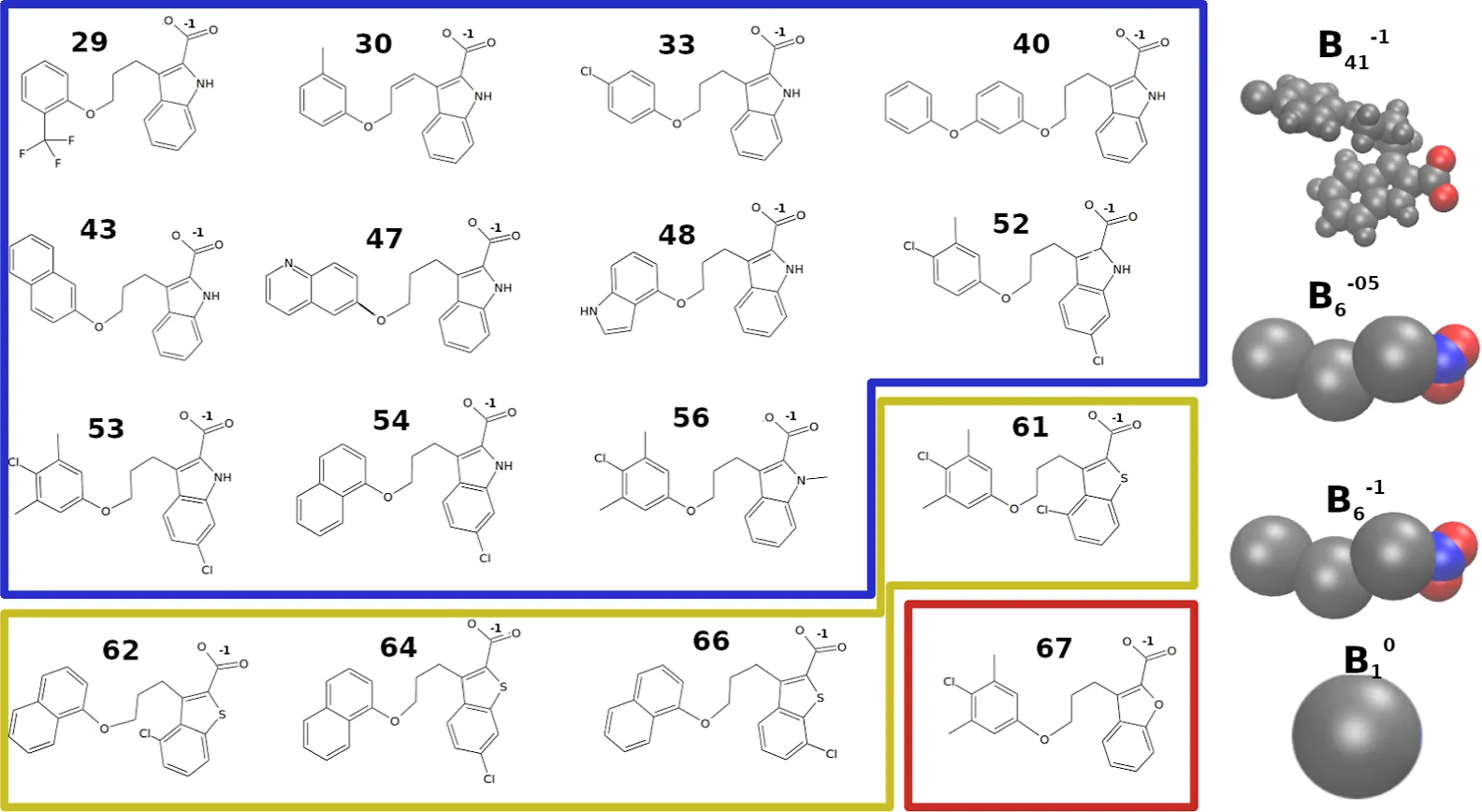
MCL-1 ligands. Scaffold core families are boxed in blue (indole), yellow (benzothiophene), and red (benzofuran). LJ blobs are depicted on the right: blue, red, and gray indicate positively charged, negatively charged, and neutral atoms, respectively. The subscript and superscript in the blobs refer to the number of atoms and the total charge, respectively.

We present here results for RBFEs obtained using four different LJ blobs in our DT-NE scheme (see Figure 3). In the Zenodo repository (https://zenodo.org/records/21418117), results using up to seven different blobs are reported (see documentation therein). Further methodological details on ligand and blob modeling, system preparation, HREM setup, and the NE-DT alchemical protocol are reported in the Supporting Information.

### 3.2 HREM Sampling of the Bound State

As stated in the Theory section, while end-state blob sampling poses no difficulties because of their conformational rigidity, limited size, and force field simplicity, the HREM sampling of the end-states of the fully coupled ligand in the complex and in bulk is crucial for the DT-NE alchemical protocol. The mean acceptance ratio across the 16-replica 30 ns simulations of the MCL-1-ligand complexes was around 40%, with an average round-trip time of a few ns. For the 6-replica 16 ns simulations of the real ligands in bulk solvent, the mean acceptance ratio and round-trip time were 30% and 0.15 ns, respectively. Full details on the HREM simulations (definition of the “hot region” in the state Hamiltonians, scaling protocol, exchange frequency) are reported in the Supporting Information.

In Figure 4, we show the equilibrium probability distributions of the COM–COM distance of the four blob-MCL-1 complexes compared to the cumulative distribution across all ligands’ COM–COM distances in the target state. The MCL-1 congeneric ligands exhibit a common well-defined pose with a maximum at $R_{COM-COM}≃10.5Å$ and a secondary pose at $R_{COM-COM}≃13Å$ . Note that the COM–COM cumulative distribution of the hottest replica (scaling factor 0.2, corresponding to a “temperature” of 2000 K) is significantly broadened and shifted toward larger distances. Thus, in the “high-temperature” states, the HREM simulation samples poses that differ significantly from the starting (presumably optimal^49^) pose. Nonetheless, despite the regular diffusion exhibited by replicas across all HREM states as measured by the acceptance exchange ratio and the replica flux,^61^ only a tiny fraction of these suboptimal poses is transmitted via replica exchanges to the target state, confirming that the starting pose is close to a conformational free energy minimum for all 16 ligands.

**Figure 4:**
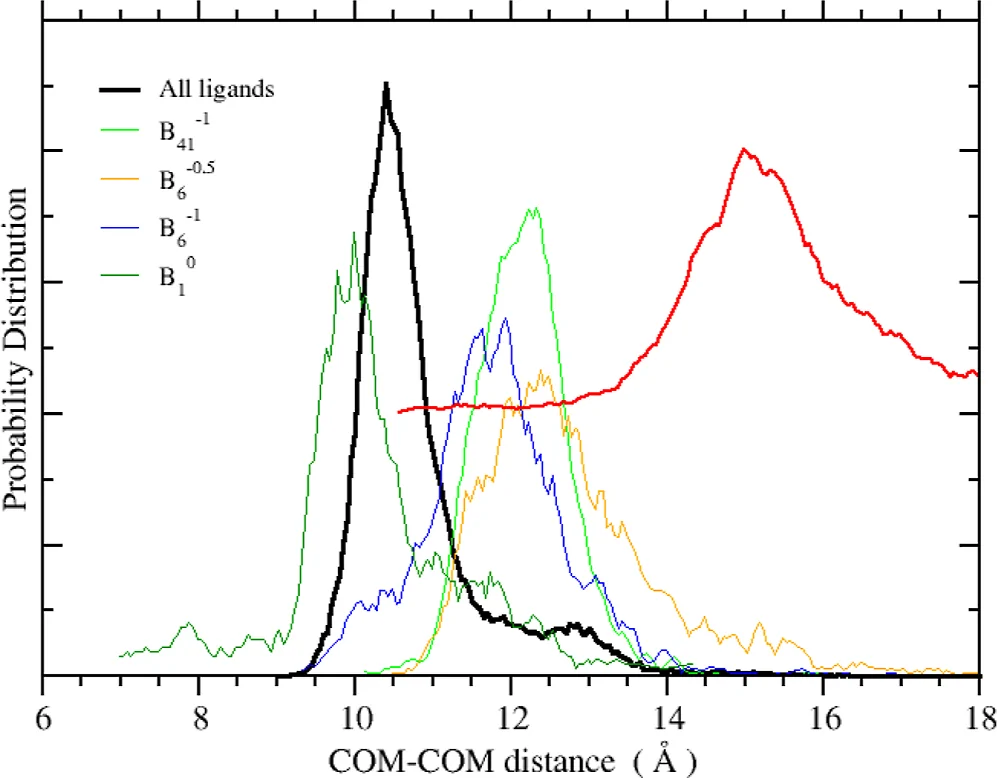
Probability distribution of the distance between the protein and ligand (or blob) centers of mass. In thick black and red, the cumulative distribution for the 16 MCL-1 ligands in the target state and in the “hottest” HREM state. The COM–COM distributions for the various blob–protein complexes are also shown. ABCG2 charges were used for the ligands.

Regarding sampling of the blobs, Figure 4 shows that the COM–COM distributions of the blobs are wider compared to those of the ligands, revealing a moderate to pronounced mobility of the blobs inside the MCL-1 binding site. The maximum of the B_41_^–1^, B_6_^–0.5^, and B_6_^–1^ COM–COM distributions is shifted, to various degrees, toward the position of the secondary pose. The distribution of the simple B_1_^0^, while significantly overlapping with the distributions of the real ligands, displays maximum mobility, with *R*_COM–COM_ extending from 7 to 14 Å. As in the initial bound state of the blob → *L* transition, the COM of ghost ligand *L* is made coincident with that of the fully coupled blob (see Figure S1 and related discussion in the Supporting Information for details on how the HREM sampled conformations of the individual ligands in the bound state and in the gas-phase are joined together to construct the dual-topology alchemical system), during the NE alchemical switch in the complex, the recoupling *L* ligand must mechanically drag the decoupling blob toward the stable pose at full *L* coupling. As shown in Figure S2 and Table S1, this “dragging”, while not affecting the RBFE,^37^ generally implies large dissipation. Dissipation is less critical for the *L* → blob bound state transition (see Figure S2), thus affording a reliable BAR-based crossing point determination of the forward and reverse work distributions in all cases.

### 3.3 RBFE with the Blob Reference

In Table 1, we report the RBFE for the *L*_*i*_ → blob transformation for the four blobs of Figure 3, determined using vDSSB-BAR (see eq 6 and Methods Section in the Supporting Information).

**Table 1:** BAR Estimates (in kcal/mol) for the *L*_*i*_ → Blob RBFE Using the Four Blobs Reported in Figure 3 ^a^

*L*	B_41_^–1^	B_6_^–0.5^	B_6_^–1^	B_1_^0^
29	–2.80 ± 0.60	8.92 ± 0.33	6.18 ± 0.42	5.87 ± 0.41
30	–4.69 ± 0.33	8.98 ± 0.34	5.68 ± 0.30	5.07 ± 0.37
33	–2.56 ± 0.32	8.77 ± 0.29	5.81 ± 0.25	5.10 ± 0.34
40	–3.55 ± 0.48	9.54 ± 0.44	6.47 ± 0.39	5.98 ± 0.41
43	–2.71 ± 0.64	8.57 ± 0.24	6.03 ± 0.38	5.08 ± 0.56
47	–5.54 ± 0.64	6.62 ± 0.32	3.85 ± 0.35	3.25 ± 0.50
48	–7.00 ± 0.44	5.87 ± 0.28	2.57 ± 0.33	2.24 ± 0.32
52	0.91 ± 1.00	11.78 ± 0.42	9.45 ± 0.45	8.79 ± 0.60
53	0.28 ± 0.51	12.60 ± 0.51	9.58 ± 0.41	9.92 ± 0.56
54	0.00 ± 0.64	12.95 ± 0.59	9.38 ± 0.54	8.60 ± 0.28
56	–0.17 ± 0.76	12.37 ± 0.37	9.25 ± 0.44	9.50 ± 0.52
61	–0.58 ± 0.37	12.36 ± 0.34	8.62 ± 0.29	7.83 ± 0.58
62	–1.40 ± 0.70	11.26 ± 0.49	7.80 ± 0.34	7.14 ± 0.52
64	–1.87 ± 0.51	11.94 ± 0.37	9.03 ± 0.51	8.16 ± 0.78
66	–0.96 ± 0.52	13.10 ± 0.68	10.39 ± 0.49	9.50 ± 0.77
67	–0.29 ± 0.53	11.91 ± 0.33	8.56 ± 0.31	7.67 ± 0.50
B_6_^–1^	-	2.60 ± 0.14	-	–0.58 ± 0.18

a The ABCG2 model was used for the MCL-1 ligands. The last row gives the direct RBFE between blobs (B_6_^–1^ → B_6_^–0.5^ and B_6_^–1^ → B_1_^0^).

With the exception of B_41_^–1^, all the RBFEs are positive, meaning that the binding affinities of B_6_^–0.5^, B_6_^–1^, and B_1_^0^ are consistently smaller than those of the ligands. B_41_^–1^, retaining the chemical structure of ligand 29, binds more strongly than most ligands, thus exhibiting a large absolute dissociation free energy. The blob-based RBFEs, despite referring to *independent* NE-DT calculations with different blob references bearing different charges, show a strong linear relationship, as shown in Table 2, where we report the correlation metrics between the sets of RBFEs computed for all six possible pairs, as measured by the Pearson correlation coefficient ρ, the slope of the best fitting line, the Kendall rank coefficient τ, and the mean signed deviation (MSD). Full correlation data among the RBFEs using all the seven tested blobs are reported in the Zenodo repository (https://zenodo.org/records/21418117).

**Table 2:** Correlation Metrics between Sets of Blob-Based RBFEs for the MCL-1 Ligands

	ρ	slope	τ	MSD
B_41_^–1^B_6_^–0.5^	0.93	0.98	0.63	–12.54
B_41_^–1^B_6_^–1^	0.94	0.99	0.72	–9.50
B_41_^–1^B_1_^0^	0.93	0.95	0.72	–8.91
B_6_^–0.5^B_6_^–1^	0.99	0.97	0.82	3.04
B_6_^–0.5^B_1_^0^	0.97	0.97	0.81	3.62
B_6_^–1^B_1_^0^	0.99	1.00	0.92	0.58

The metrics reported in Table 2 convincingly confirm that the choice of the reference blob, irrespective of its shape and total charge, strictly preserves correlation and ranking. The linear relationship is nearly perfect between the B_1_^0^ and B_6_^–1^ RBFE sets. Had the DT-NE method depended critically on the chemistry or topology of the reference, changing the blob would have produced nonlinear distortions, degraded rankings, and inconsistent RBFE differences. None of these effects are observed. These results demonstrate that the DT-NE/LJ-blob framework is robust and does not rely on ligand congenericity or common-core atom mappings, in contrast to conventional ST approaches.

Notably, the MSD, as discussed in the Theory section, is strictly related to the difference between the blob’s absolute binding free energies. For example, the B_41_^–1^ – B_6_^–0.5^ MSD should correspond, up to a charge-related constant, to the ABFE difference Δ*G*(B_41_^–1^) – Δ*G*(B_6_^–0.5^). It then follows that for any blob triplet *i*, *j*, *k*

13 $${MSD}_{ij}+{MSD}_{jk}+{MSD}_{ki}=0$$

must hold. Remarkably, this relation is satisfied in all cases, yielding an average ϵ_*ijk*_ = MSD_*ij*_ + MSD_*jk*_ + MSD_*ki*_ of less than 0.05 kcal/mol over the six blob triplets. This can be considered a sort of cumulative cycle closure relation and represents a stringent thermodynamic consistency check of the DT-NE protocol with the blob reference.

In Figure S3 of the Supporting Information, we also report the variance of the forward *L*_*i*_ → blob and reverse blob → *L*_*i*_ transmutations in the bound and unbound states. Since the variance of the work distribution is strictly related to the dissipation, Figures S2 and S3 show that the uncertainty in the vDSSB-BAR estimate is dominated by the highly dissipative blob → *L*_*i*_ transmutation in the bound state, i.e., the NE process consistently exhibiting the largest variance across all blob-to-ligand transmutations. In the blob → *L*_*i*_ bound state transition, although the initially decoupled ligand is located at the binding site with its COM tethered to that of the blob, its orientation and conformation (sampled from a HREM gas-phase simulation, see Methods Section 1.2 in the Supporting Information) are not adjusted to the binding pocket shape, and must optimally adapt to the protein environment while growing. While the combined effect of the shifted potential of eq 4 and the partial void sensed by the ghost *L*_*i*_ (which does not interact with the decoupling blob partner in the binding site) effectively tame the detrimental impact on dissipation of potential steric clashes in the initial stages of the alchemical transition, the structural reorganization of the growing ligand may nevertheless follow dissipative paths leading to suboptimal final poses and large work values, thus widening the work distribution. On the other hand, in the reverse process, i.e., *L*_*i*_ → blob, it is now the turn of the blob to grow in the binding site. In this case, due to the simplicity of the structural and force field features of the blob (with the exception of B_41_^–1^), the risk of steric clashes and highly dissipative paths during the growth of the blob—protected from steric hindrances by the initially coupled bulky partner—is significantly reduced, leading to relatively modest dissipation (see Figure S3).

These concepts are illustrated in Figure 5, where we report, as an example, the *L*_*i*_ → blob and blob → *L*_*i*_ vDSSB work convolutions for ligand 62 (modeled with ABCG2) with the bulky B_41_^–1^ blob (Figure 5b) and with the simple six-atom B_6_^–1^ blob (Figure 5a). For the vDSSB convolution of *P*_b_(*W*|*F*) and *P*_u_(*W*|*R*) (see eq 6), the variance of the resulting distribution is given by the sum of the variances of the bound and unbound state distributions, i.e., σ_vDSSB_^2^ = σ_b_^2^(*F*) + σ_u_^2^(*R*). As it can be seen from Figure 5, the crossing point of the forward and reverse work distributions is much sharper for the small blob B_6_^–1^ compared to that obtained with the bulky blob B_41_^–1^. The structural simplicity of the reference blob is thus a key feature for the success of the DT-NE approach. Full results for the vDSSB distributions involving all ligands and blobs are reported in the Zenodo repository (https://zenodo.org/records/21418117).

**Figure 5:**
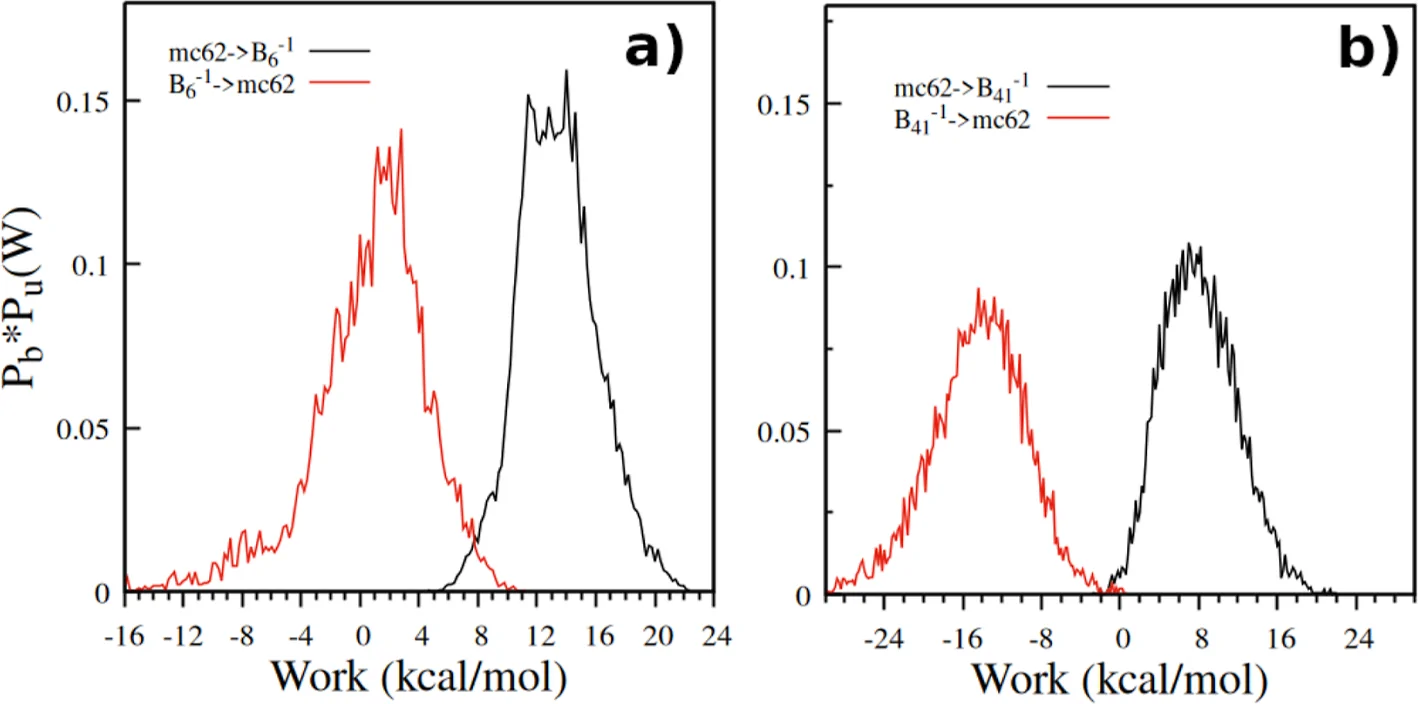
Forward and reverse vDSSB work distributions for the blob-ligand transmutations (ligand 62, ABCG2 charges) using B_6_^–1^ (a) and B_41_^–1^ (b).

### 3.4 Cycle Closure Conditions

We have verified the CCC (eq 9) for the blobs B_6_^–1^, B_6_^–0.5^, and the simple LJ blob B_1_^0^. All these blobs, unlike B_41_^–1^, yield an average confidence interval for the *L*_*i*_ → blob RBFE below 0.5 kcal/mol (see Table 1).

In Table 3, we report the calculated three-edge free energies ϵ­(*L*)—expected to be close to zero and in any case well below the total error (Err) obtained by summing in quadrature the errors in each of the three edges of the cycle (see eq 9) having as vertices one ligand and two blobs. The CCC is verified in all cases, with the exception of the cycle 61 → B_6_^–1^ → B_6_^–0.5^ → 61, for which the corresponding ϵ slightly exceeds the confidence interval. Table 3 represents a powerful and stringent test for the validation of the DT-NE approach using simple LJ blobs, whether bearing a charge or not.

**Table 3:** CCC Conditions for the *L*_*i*_ → blob1 → blob2 → *L*_*i*_ Cycles (Reported Values are in kcal/mol)^a^

cycle	ϵ(*L*)	err	cycle	ϵ(*L*)	err
29 → B_6_^–1^ → B_6_^–0.5^ → 29	–0.12	1.10	29 → B_6_^–1^ → B_1_^0^ → 29	0.31	1.15
30 → B_6_^–1^ → B_6_^–0.5^ → 30	–0.70	0.93	30 → B_6_^–1^ → B_1_^0^ → 30	0.61	0.93
33 → B_6_^–1^ → B_6_^–0.5^ → 33	–0.36	0.80	33 → B_6_^–1^ → B_1_^0^ → 33	0.71	0.83
40 → B_6_^–1^ → B_6_^–0.5^ → 40	–0.47	1.18	40 → B_6_^–1^ → B_1_^0^ → 40	0.49	1.11
43 → B_6_^–1^ → B_6_^–0.5^ → 43	0.07	0.94	43 → B_6_^–1^ → B_1_^0^ → 43	0.95	1.33
47 → B_6_^–1^ → B_6_^–0.5^ → 47	–0.17	0.97	47 → B_6_^–1^ → B_1_^0^ → 47	0.60	1.20
48 → B_6_^–1^ → B_6_^–0.5^ → 48	–0.70	0.89	48 → B_6_^–1^ → B_1_^0^ → 48	0.33	0.90
52 → B_6_^–1^ → B_6_^–0.5^ → 52	0.27	1.24	52 → B_6_^–1^ → B_1_^0^ → 52	0.66	1.63
53 → B_6_^–1^ → B_6_^–0.5^ → 53	–0.15	1.21	53 → B_6_^–1^ → B_1_^0^ → 53	–0.34	1.36
54 → B_6_^–1^ → B_6_^–0.5^ → 54	–0.97	1.59	54 → B_6_^–1^ → B_1_^0^ → 54	0.78	1.19
56 → B_6_^–1^ → B_6_^–0.5^ → 56	–0.52	1.16	56 → B_6_^–1^ → B_1_^0^ → 56	–0.25	1.34
61 → B_6_^–1^ → B_6_^–0.5^ → 61	–1.14	0.92	61 → B_6_^–1^ → B_1_^0^ → 61	0.79	1.63
62 → B_6_^–1^ → B_6_^–0.5^ → 62	–0.86	1.20	62 → B_6_^–1^ → B_1_^0^ → 62	0.66	1.22
64 → B_6_^–1^ → B_6_^–0.5^ → 64	–0.31	1.27	64 → B_6_^–1^ → B_1_^0^ → 64	0.87	2.17
66 → B_6_^–1^ → B_6_^–0.5^ → 66	–0.11	1.67	66 → B_6_^–1^ → B_1_^0^ → 66	0.89	1.96
67 → B_6_^–1^ → B_6_^–0.5^ → 67	–0.75	0.93	67 → B_6_^–1^ → B_1_^0^ → 67	0.89	1.15

a The mutual RBFEs between B_6_^–1^ and B_6_^–0.5^, and between B_6_^–1^ and B_1_^0^, are listed in the last row of Table 1.

### 3.5 Blob-Based DT-NE RBFEs vs Experiment

The *L*_*i*_ → blob RBFEs of Table 1 for the 16 MCL-1 ligands, besides yielding the ABFEs up to a constant related to the common blob ABFE, *give immediate access to all possible 120 RBFEs* between real ligands using eq 5 via two-edge transformations of the form *L*_*i*_ → blob → *L*_*j*_.

In Table 4 we report ρ, τ, slope, and MUD obtained by comparing the computed RBFEs using the DT-NE approach and ABCG2 to the experimental counterparts. In the table, we also report the metrics for the 120 RBFEs obtained in ref 49 using ST-NE alchemy and the ABCG2 model. Note that in ref 49 only 29 RBFEs between the same 16 ligands were determined by one-edge ST transmutations due to the limitation of the ST approach to sufficiently similar (congeneric) ligands. The remaining 91 RBFEs have been determined from the Behera et al.^49^ data using two-edge or three-edge transmutations involving intermediate ligands. The comparison of ST RBFEs and DT-NE is not strictly equivalent and may actually favor the present DT-NE/blob methodology, since the statistical uncertainties associated with multi-edge ST perturbations accumulate along the perturbation path. For complete transparency, the Zenodo repository https://zenodo.org/records/21418117 includes the Python scripts used to reconstruct the 120 ST-NE RBFEs from the original Behera data set github.com/deGrootLab/abcg2_evaluation/, and in Table 4, we also report the metrics for the complete set of 123 direct single-edge ST-NE calculations reported in ref 49 for the entire MCL-1 ligand series (spanning approximately 50 ligands).

**Table 4:** Correlation Metrics between Experiment^60^ and Blob-Based RBFEs for 120 RBFEs (Computed with the ABCG2 Model) Involving the 16 MCL-1 Ligands of Figure 3 ^a^

type	ρ	τ	MUD	*n*
B_41_^–1^	0.71	0.63	1.61	120
B_6_^–0.5^	0.81	0.63	1.68	120
B_6_^–1^	0.81	0.63	1.52	120
B_1_^0^	0.84	0.65	1.54	120
ST(a)	0.78	0.58	1.35	120
ST(b)	0.73	0.54	1.36	123

a MUD is in kcal/mol. The entry “ST­(a)” refers to the 120 RBFEs obtained with the ST approach^49^ on the 16 MCL-1 using one to three edge transformations ligands. The entry ST­(b) refers to all 123 single-edge RBFEs reported in ref 49. ABCG2 atomic charges for the ligands were used.

The metrics referring to the DT-NE blob-based calculation are generally satisfactory and similar for all blobs, demonstrating the consistency and robustness of our methodological approach. The agreement with the experimental values^60^ obtained using the blob references B_6_^1–^ and B_1_^0^ is excellent and similar to, if not better than, that obtained with the ST approach. Interestingly, even under the DT/ST asymmetric comparison for the 120 RBFEs among the 16 MCL-1 ligands, the ST are actually better than those obtained on the complete set of direct ST transformations (see last entry in Table 4.)

The ability to reconstruct the complete 120-entry RBFE matrix from only 16 independent blob-ligand calculations, while maintaining agreement with the corresponding experimental and ST-RBFE results, constitutes a further stringent test of the internal thermodynamic consistency and robustness of the proposed approach.

In Figure 6, we show the correlation plots between experimental RBFE and the calculated RBFE using B_6_^–1^ and B_1_^0^, i.e., the reference blobs exhibiting the best metrics in Table 4. Error bars for the two-edge blob-based RBFEs are not shown for the sake of clarity; they can be deduced from Table 1 by summing in quadrature the 95% confidence intervals reported for the *L*_*i*_ → blob transitions.

**Figure 6:**
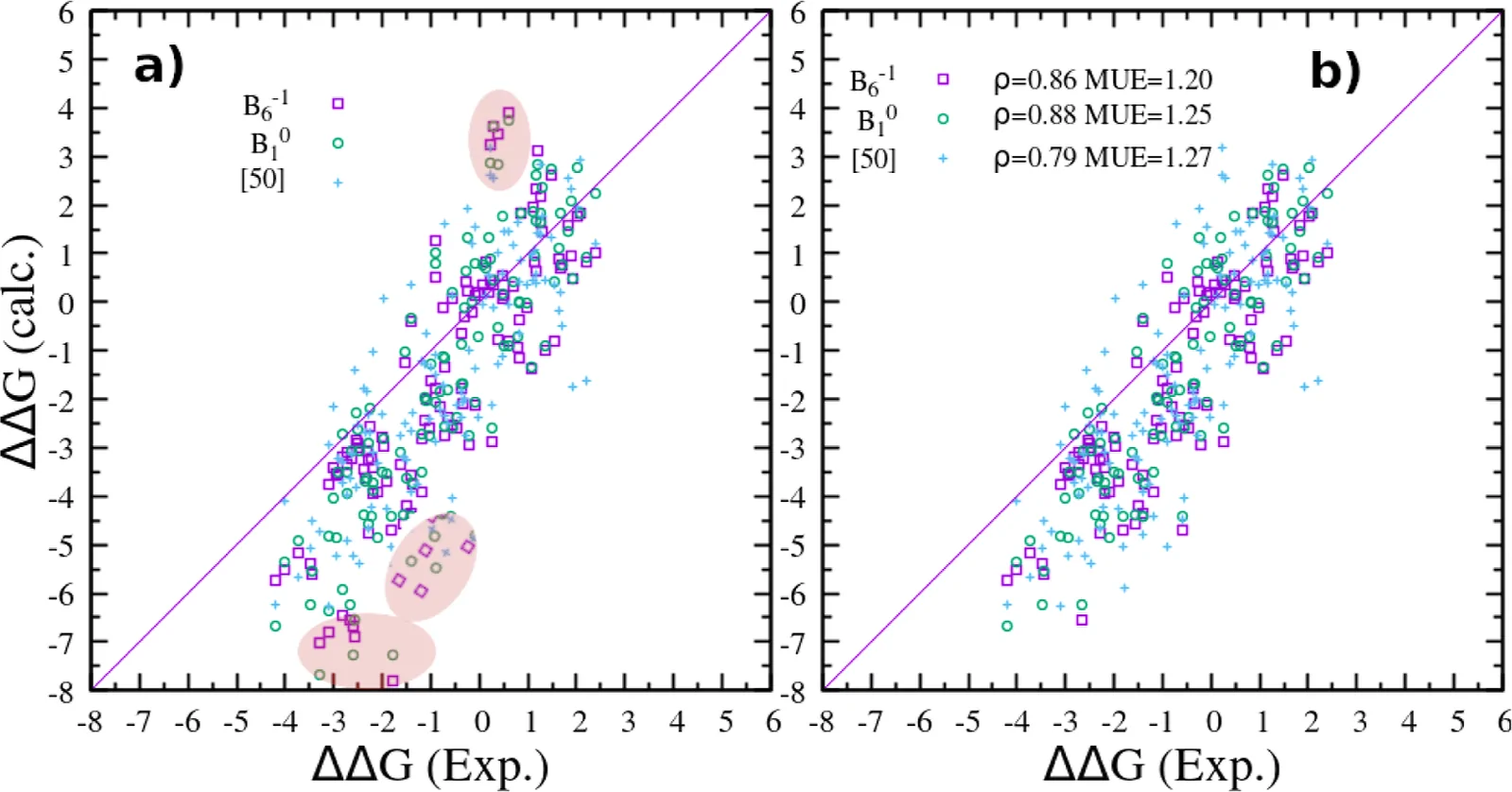
Correlation plot between experimental and computed RBFEs (units of kcal/mol) using DT-NE (squares and circles) and ST-NE (crosses) including (a) and excluding (b) RBFEs involving ligand 48. Ligand 48-related outliers are highlighted in the shaded areas in (a). ABCG2 atomic charges for the ligands were used.

Some data appear to cluster off the best-fitting line, both when using B_6_^1–^ and B_1_^0^ reference blobs (see Figure 6a). The corresponding RBFEs systematically involve ligand 48 for both B_6_^–1^ and B_1_^0^ reference blobs. If this ligand is removed from the correlation data, the ρ and τ further improve, hitting, for the simplest LJ blob B_1_^0^, values of 0.88 and 0.89, respectively, with the MUD dropping to ≃1.25 kcal/mol. On the other hand, by removing data involving ligand 48 from the Behera et al.^49^ RBFEs, the improvement in the correlation metrics is less significant. The strong linear relationship for RBFEs computed with disparate blobs (see Table 2), besides confirming the robustness and reproducibility of the DT-NE approach with the reference blob, may help to identify *force-field-induced* outliers (vide infra).

### 3.6 AM1-BCC/ABCG2 Comparison

AM1BCC and ABCG2 both use GAFF2 atomic types^62^ and differ only in the bond charge correction (BCC) to common AM1 Mulliken charges.^48^ In the ABCG2 model, BCCs have been extensively reoptimized for specific functional groups against high-quality experimental hydration free energy data. In Figure 7 we show the correlation plot between the AM1BCC and ABCG2 atomic BCC charges including all 735 atoms in the 16 MCL-1 ligands considered in this study.

**Figure 7:**
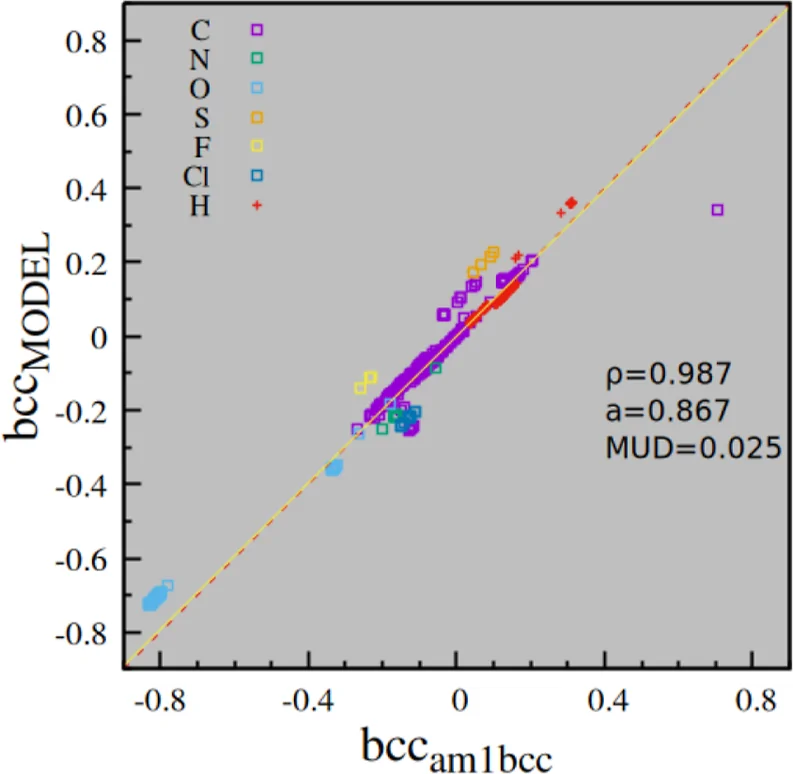
AM1BCC-ABCG2 atomic BCC (in e units) correlation for all atoms in the ligands of Figure 3. The red dashed line indicates perfect correlation; the yellow line is the best-fitting line.

As can be seen, BCCs are strongly correlated for the two models, yielding an MUD of only 0.025 e. Notable outliers are fluorine atoms in ligand 29, whose BCC charges are significantly less negative in the ABCG2 model. Correspondingly, the positive BCC of the carbon atom in the CF_3_ group is much larger with ABCG2 than with AM1BCC. Thus, based on Figure 7, we do not expect large discrepancies between the AM1BCC and ABCG2 RBFEs computed with the DT-NE approach. This is fully confirmed by comparing the AM1BCC and ABCG2 120 RBFEs, computed using the reference blobs B_6_^–1^ and B_1_^0^: we obtain a ρ of 0.94 and τ of 0.75 in both cases, and an MUD below 1 kcal/mol, with the best-fitting line slope of 1.01 and 1.14 for B_6_^–1^ and B_1_^0^, respectively. The corresponding correlation plots are reported in the Supporting Information, Figure S4.

In Table 5, we show the metrics obtained by comparing the DT-NE AM1BCC and ABCG2 RBFEs using the blobs B_6_^–1^ and B_1_^0^ with the experimental data. In the table we also show the metrics obtained on the same set of MCL-1 RBFEs using the ST approach.^49^

**Table 5:** Correlation Metrics between Experimental and Calculated Data (Using NE-DT Alchemy) for the 120 RBFEs among the 16 Ligands of Figure 3 ^a^

	ρ		τ		MUD (kcal/mol)		
	AM1BCC	ABCG2	AM1BCC	ABCG2	AM1BCC	ABCG2	*n*
B_6_^–1^	0.83	0.81	0.61	0.63	1.32	1.52	120
B_1_^0^	0.79	0.84	0.55	0.65	1.49	1.54	120
ST	0.78	0.79	0.58	0.59	1.34	1.71	120
B_6_^–1^	0.86	0.86	0.63	0.67	1.10	1.20	105
B_1_^0^	0.79	0.88	0.55	0.68	1.41	1.22	105
ST	0.79	0.80	0.59	0.59	1.27	1.50	105

a The last three rows refer to the 105 RBFEs obtained by excluding the RBFEs involving ligand 48. Entries marked “ST” refer to the results obtained from the Behera et al. data.^49^

Differences are in general small and statistically not significant, with ABCG2 exhibiting marginally better ranking and correlation but slightly worse MUD in three cases out of four. For the ABCG2 model, DT-NE seems to perform slightly better than ST-NE calculations from Behera et al.^49^ in correlation, ranking, and MUD. The average MSD and MUD of the RBFEs involving ligand 29 obtained with the AM1BCC and ABCG2 models using DT-NE are similar and small (MUD ≃ 0.9–1 kcal/mol and MSD ≃ −0.1 to +0.2 kcal/mol), suggesting that the cited differences in BCC charges on the CF_3_ group do not affect significantly the computed RBFEs. Remarkably, by excluding the ligand 48 RBFEs from the sets, we consistently notice a significant improvement in the ABCG2 metrics, irrespective of the reference blob and of the NE alchemical approach, hence confirming an ABCG2-induced force field bias for ligand 48.

### 3.7 Predicted ABFE of the 16 MCL-1 Ligands

As previously discussed, ABFEs, up to a blob-dependent constant offset, *are already available from* Table 1 so that correlation and ranking vs experiment can be immediately assessed. The constant offset—which is independent of the adopted model—is nothing other than the ABFE of the blob itself.

However, if even a single experimental ABFE is available for the ligand set, there is no need to compute the blob ABFE explicitly. If ligand *L*_*i*_ has an experimental binding free energy (Δ*G*_*i*_^exp^), then all ligand ABFEs are immediately obtained through

14 $$\Delta G_{j}^{0}=\Delta \Delta G\left(blob\to L_{j}\right)+c_{i}c_{i}=\Delta G_{i}^{\exp}-\Delta \Delta G\left(blob\to L_{i}\right)$$

Thus, one experimental ABFE is sufficient to anchor the entire blob-based RBFE network.

Nevertheless, the methodology is also capable of predicting ABFEs in the absence of experimental data by using eq 8. To this end, we use blob B_6_^–1^ as the universal reference. This choice is motivated by the fact that this blob has the same charge of the MCL-1 ligands (thereby avoiding the excess dissipation due to charge annihilation), by its comparatively limited mobility within the MCL-1 binding pocket (see Figure 4), which produces narrower bound-state work distributions and consequently more precise ABFEs estimates. The ABFEs are therefore evaluated as

15 $$\Delta G_{i}^{0}=\Delta G^{{}^{\circ}}\left(B_{6}^{-1}\right)+\Delta \Delta G\left(B_{6}^{-1}\to blob\right)+\Delta \Delta G\left(blob\to L_{i}\right)$$

where ΔΔ*G*(blob → *L*_*i*_) is the RBFE between the selected blob and ligand *L*_*i*_, ΔΔ*G*(blob → B_6_^–1^) is the relative binding free energy between the selected blob and the reference blob, B_6_^–1^, obtained from the average shift of the corresponding blob–blob correlations (see MSD column in Table 2), and Δ*G* ^°^(B_6_^–1^) is the vDSSB absolute binding free energy of the reference blob. The reference B_6_^–1^ ABFE has been calculated using the vDSSB approach for unidirectional estimates.^44^ Since BAR is not used, to increase precision and accuracy we used 288 NE trajectories for the bound state instead of 96 (as in RBFE BAR-based calculations).

In Figure 8 we report the experimental ABFEs of the 16 MCL-1 ligands against the B_1_^0^-related and B_6_^–1^-related ABFEs using both AM1BCC and ABCG2 models, computed via eq 14 with the Δ*G*_*i*_^exp^ of ligand 29 as reference (Figures 8a,b) and via the fully predictive eq 15 (Figures 8c,d).

**Figure 8:**
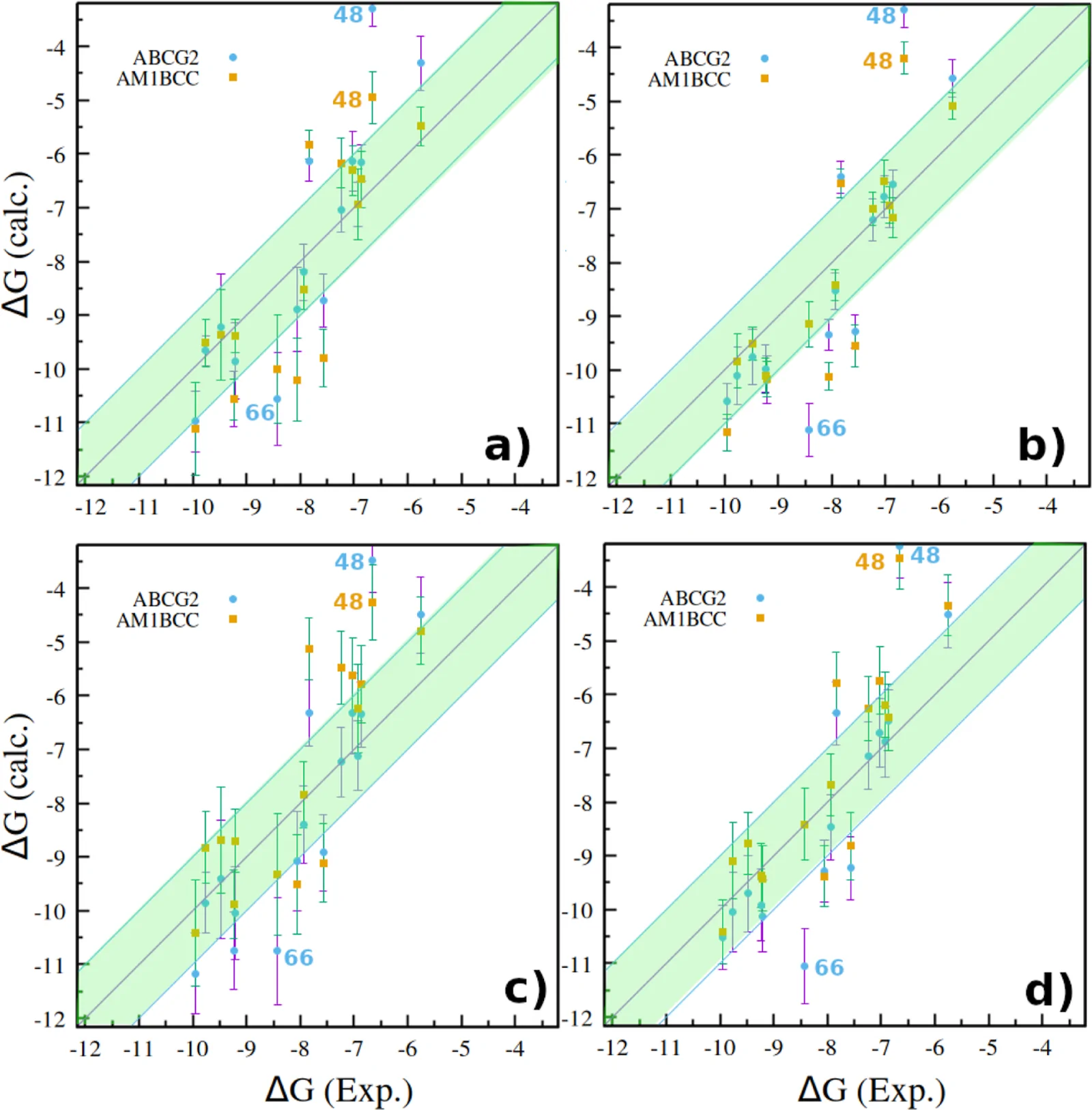
Binding free energies (in kcal/mol) for the 16 MCL-1 ligands (kcal/mol) computed using the eq 14 (a,b) and eq 15 (c,d). (a,c) refer to B_1_^0^-based ABFEs. (b,d) refer to B_6_^–1^-based ABFEs. The straight line indicates perfect correlation. The shaded green area marks MUD within 1 kcal/mol.

As for the method provided by eq 14, note that the constant offset *c*_*i*_ in eq 14 implicitely includes both the charge- or volume-related corrections. The quality of the agreement, on the other hand, depends strongly on the choice of the particular selected ligands *i*, as any force field bias on ΔΔ*G*(blob → *L*_*i*_) is propagated to all other ABFEs. For example, if we select ligand 48 instead of ligand 29 (a clear outlier in ABCG2, see Figure 6) as the anchor for experimental free energy, the MUD in the ABFEs raises to 2.6 kcal/mol. The *methodological* uncertainty of each predicted ABFE according to eq 14 is simply obtained by propagating the uncertainties of the two corresponding blob-RBFE calculations. In the Zenodo repository https://zenodo.org/records/21418117, we provide the scripts “abs_ref 2.bash” to compute the ABFEs and metrics according to eq 14 using, in turn, the experimental free energies of all 16 MCL-1 ligands. In retrospective studies, eq 14 can hence be used as a powerful test to isolate force field biases from methodological errors on chemically diverse ligands.

While ranking and correlation between the two methodologies for ABFE calculations is identical by design, ABFEs computed according eq 15 (Figures 8c,d) consistently exhibit wider uncertainties. The larger confidence intervals observed in Figures 8c,d originate from the uncertainty associated with the vDSSB estimate of the reference blob (B_6_^–1^) ABFE, which is propagated to all ligand ABFEs together with the uncertainties of the two relative free-energy terms. Consequently, the fully predictive protocol inevitably exhibits larger confidence intervals than the experimentally anchored approach. We finally recall that the reported ABFEs in eqs 15 or 8 are defined up to a constant which is, in principle, unrelated to the blob (see Section 3.3). Such a constant, common to all MCL-1 ligands, arises from the charge correction and the binding site volume correction to the binding affinities, which are both small and *positive*. The former can be evaluated from the 800 Å^3^ volume (see Methods Section in the Supporting Information) spanned by the imposed flat-bottom potential in the bound state,^16,18^ while the latter—involving changes of 1 e units—can be standardly computed as a postcorrection,^26,63–65^ yielding a total combined offset of ≃+0.5 kcal/mol.

Note that, using ABCG2, besides the aforementioned ligand 48, also ligand 66 emerges as an outlier (Δ*G* ^calc^ – Δ*G* ^exp^ > 2 kcal/mol) in the ABFE calculations with both eqs 14 and 15.

In Table 6, the salient metrics for calculated-experimental ABFEs are shown. The blob-dependent correlation, ranking and slope of the best fitting line are identical for the two ABFE methodologies within a given force field model, as the corresponding ABFEs only differ by the blob-dependent offset. Both force field models exhibit a slope greater than 1 irrespective of the reference blob, indicating that both force fields tend to overestimate the ABFE *differences*. Despite the strong correlation for blob-ligand RBFEs (see Table 2), and despite the fact that the B_6_^–1^- and B_1_^0^-based ABFEs differ, in principle, by a *common offset* related to the binding site volume and charge corrections of the ligands, the results for the MUD are consistently similar across the two blob-related ABFEs, the two force fields and the two methodologies. In conclusion, ABCG2 and AM1BCC ABFEs show similar performance in terms of ranking, correlation, MUD and MSD (see Table 6), hence confirming the recent results obtained in ref 49 concerning the AM1BCC-ABCG2 comparison for the MCL-1 ligands.

**Table 6:** Correlation Metrics between Blob-Based ABFEs and Experimental ABFEs for the MCL-1 Ligands^a^

	eq 14									
	AM1BCC					ABCG2				
	ρ	τ	*a*	MUD	MSD	ρ	τ	*a*	MUD	MSD
B_6_^–1^	0.86	0.65	1.42	0.87	0.22	0.85	0.72	1.55	0.99	0.17
B_1_^0^	0.82	0.58	1.37	0.98	0.17	0.87	0.73	1.61	1.00	–0.08
Equation 15										
B_6_^–1^	0.86	0.65	1.42	0.95	–0.52	0.85	0.72	1.55	0.98	0.11
B_1_^0^	0.82	0.58	1.37	1.15	–0.52	0.87	0.73	1.61	1.01	0.11

a *a* denotes the slope of the best-fitting line.

## 4 Discussion

The DT-NE/LJ-blob methodology proposed in this work belongs to the broader family of approaches developed to overcome the limitations of single-topology RBFE calculations when ligand pairs do not share a well-defined common core, atom mapping, or conserved binding pose. It is therefore useful to place the present scheme in the context of related dual-topology, separated-topology, alchemical-transfer, and reference-state methodologies.

The QligFEP workflow of Jespers et al.^34^ is one of the closest precedents in spirit. As in DT-NE, both ligands are simultaneously present with their own topologies, and one ligand is decoupled while the other is recoupled. However, QligFEP is based on equilibrium FEP stratification, whereas DT-NE uses end-state HREM sampling followed by bidirectional nonequilibrium transformations and Crooks/BAR analysis. More importantly, QligFEP maintains the alignment of selected equivalent atoms between the two ligands by applying distance restraints. These restraints are designed to keep the common substructure of the two ligands superposed during the alchemical transformation. This strategy is effective when a meaningful common core exists, but it implies that the method is not fully pose-agnostic: the relative orientation of the shared substructure is imposed by construction. In contrast, in DT-NE the two molecules are connected only through a COM–COM tether in the bound state. Since the ghost molecule is completely noninteracting, this restraint does not impose any atom-pair alignment, common-core overlap, or orientation of one ligand relative to the other. The end-state configurational distribution therefore remains that of the physical ligand in the binding site, while the ghost molecule only provides the topology needed for the subsequent nonequilibrium transformation.

A related strategy is the hybrid single-/dual-topology method of Jiang et al.^32^ In this case, matching atoms in the common substructure are connected through holonomic constraints rather than harmonic restraints. This removes some of the complications associated with finite force-constant restraint energies, but the method still relies on the existence of equivalent atoms and on a predefined alignment of the common molecular core. Consequently, the approach remains tied to transformations in which a meaningful core-mapping can be identified. As in QligFEP, different orientations or shifted poses of the common substructure at the two physical end-states are not naturally accommodated. DT-NE avoids this assumption by not requiring atom matching at all. The two end-states are sampled independently, and the dual-topology system is assembled only for the nonequilibrium switching step.

The separated-topologies approach of Baumann et al.^35^ is probably the closest methodology to DT-NE at the thermodynamic-cycle level. In both cases, the RBFE can be viewed as the difference between two alchemical binding processes involving ligands that retain their independent topologies throughout the calculation. However, separated-topology calculations generally rely on Boresch-type restraints^66^ to anchor each ligand to the binding site. These restraints provide a rigorous route to the standard-state correction, but they also require the definition of a specific bound pose and of suitable ligand–protein anchor atoms. The method is therefore not completely pose-agnostic and may require nontrivial system-dependent choices, particularly for ligands with multiple binding modes or diffuse binding regions. In DT-NE, by contrast, the equilibrium end-state sampling is performed without imposing orientational restraints on the ligand. The only bound-state tether used in the dual-topology construction is the COM–COM restraint between the physical molecule and its ghost partner; this restraint is not a positional or orientational restraint to the receptor.

Another closely related and very elegant development is the Alchemical Transfer Method (ATM) of Gallicchio and co-workers.^33^ In ATM, two noninteracting molecular representations are present in the same simulation box, one located in the binding site and the other in bulk, separated by a displacement vector. Free energies are obtained through a symmetric alchemical cycle, using a common reference state at the midpoint of the transformation. This construction avoids several difficulties of standard decoupling approaches and provides a unified framework for ABFE and RBFE calculations. Its main practical difference with respect to DT-NE is that the bound and bulk ligands coexist in the same simulation box. To avoid direct or solvent-mediated interactions between them, the displacement vector must be sufficiently large, which typically requires a large simulation box. DT-NE instead keeps the complex and solvent legs in separate systems, using a standard complex box and a smaller solvent box. Thus, the two approaches share the goal of avoiding single-topology atom mapping, but differ substantially in their statistical-mechanical implementation and computational requirements.

The present work is also conceptually related to the one-step perturbation (OSP) reference-state methodology of Oostenbrink and van Gunsteren.^42,43^ OSP introduced the idea of using an artificial reference compound to connect structurally diverse ligands. However, OSP is not an alchemical transformation method in the sense used here. It relies on equilibrium exponential reweighting from the reference ensemble using the Zwanzig formula.^14^ Consequently, the reference compound must be engineered so that its sampled configurational and energetic distributions overlap sufficiently with those of the target ligands. In practice, the OSP reference therefore preserves key structural and interaction motifs of a ligand family. In DT-NE, the reference blob plays a different role: it is an alchemical end point rather than a reweighting ensemble. It need not reproduce the pharmacophoric features, topology, or binding mode of the ligands. This distinction is illustrated by the present results, where simple LJ blobs and more elaborate ligand-derived blobs yield highly consistent blob–ligand RBFEs.

The main conceptual difference between DT-NE and the above methods is therefore not merely the use of a dual topology, but the combination of three features: independent end-state sampling, absence of atom-pair or orientational restraints between the ligands and the receptor, and bidirectional nonequilibrium switching analyzed with Crooks/BAR estimators. This makes the method intrinsically independent of common-core atom mappings and conserved binding poses. At the same time, the present results also show that the construction of the reference blob is not a trivial detail. Although rankings and RBFE correlations are remarkably robust with respect to blob size, shape, charge, and force field, the mobility and placement of the blob within the binding site affect the dissipative work and hence the statistical efficiency of the calculation. Blob design should therefore be regarded as a central methodological component of the approach.

## 5 Conclusions

In this work, we have introduced a blob-based DT-NE framework for RBFE and ABFE calculations using a receptor-specific LJ reference state. The methodology was tested on the MCL-1 benchmark, a ligand set that is deliberately favorable to conventional ST-FEP approaches because the compounds are congeneric and share a bulky common core. This therefore constitutes a stringent stress test for DT-NE, since the blob-based calculations never exploit ligand–ligand similarity, common-core atom mappings, or conserved binding poses.

The results show that ligand–blob RBFEs obtained with different reference blobs and force fields are strongly correlated, preserve ligand rankings, and yield consistent ligand–ligand RBFEs through two-edge blob-mediated transformations. The agreement with both experimental data and previously reported ST-NE results demonstrates that the DT-NE/LJ-blob approach can recover the thermodynamic structure of a ligand series even when the reference state is chemically and topologically unrelated to the ligands.

The main strengths of the method are its intrinsic parallelism, the absence of pairwise perturbation-map optimization, the use of bidirectional nonequilibrium work distributions analyzed through Crooks/BAR estimators, the availability of statistically meaningful confidence intervals, and its pose-agnostic character. These features make the approach particularly attractive for chemically diverse compound sets, where standard single-topology transformations are difficult or ill-defined.

At the same time, the present study indicates that blob construction is a central methodological issue. The development of automated, receptor-specific protocols for optimal blob design and for robust blob ABFE estimation will therefore be an important direction for future work.

We do not view DT-NE/LJ-blob alchemy as a replacement for ST-FEP, FEP+, or ST-NE methods. Rather, these approaches are complementary. Blob-based DT-NE can be used for large-scale screening of chemically diverse libraries and scaffold identification, while ST-FEP/FEP+ or ST-NE can subsequently refine congeneric series with high precision. In this combined workflow, DT-NE and ST-NE/FEP/FEP+ may also provide reliable synthetic free-energy data at scale, supporting the training of data-driven and large-language-model-based approaches for molecular design and drug discovery.

## Supplementary Material



## Data Availability

Raw work data and force field parameter files are available at the general-purpose open-access repository Zenodo (https://zenodo.org/records/21418117). A brief description of the directory tree of the repository is provided in the Supporting Information. All calculations were performed using the ORAC program^67^ on the CRESCO8 cluster, provided by ENEA.^68^ The ORAC program (v6.3) is available for download under the GPL at the Web site http://www1.chim.unifi.it/orac/.

## Notes

Methods section: system preparation, blobs’ force field, HREM simulations, DT starting configuration setup, DT-NE alchemical simulations and RBFE calculations. Dissipation in the NE-DT transition with blobs. AM1BCC/ABCG2 correlation for the two-edge RBFEs of MCL-1 ligands (PDF)
