# Supplementary material for: One Blob to Rule Them All: A Universal Dual-Topology Nonequilibrium Framework for Relative and Absolute Binding Free Energies via a Lennard-Jones Blob Reference
Source: J Chem Theory Comput. 2026 Aug 12;22(16):8627–42. doi: 10.1021/acs.jctc.6c00932 (PMC13523705; doi:10.1021/acs.jctc.6c00932)
Supplement: Supplementary file 1 [file ct6c00932_si_001.pdf]

Supporting Information for: "One Blob to Rule Them All: A Universal Dual-Topology Nonequilibrium Framework for Relative and Absolute Binding Free Energies via a Lennard-Jones Blob Reference"

Piero Procacci*

Dipartimento di Chimica "Ugo Schiff", Università degli Studi di Firenze, Via della Lastruccia 3, 50019 Sesto Fiorentino, Italy

E-mail: piero.procacci@unifi.it

1 Methods

1.1 System preparation

MCL-1 ligands were modeled using the GAFF2 force field[1] with both AM1BCC and ABCG2 atomic charges. For the MCL-1 protein, we used the AMBER99-SB-ILDN parameterization,[2] and for water the TIP3P model. We tested seven different LJ blobs in our DT-NE scheme The force field nonbonded parameters of the blobs are summarized in Table S1. Bonded terms include only stretching and bending with zero torsional potentials. Full numerical details of the blob force field parameters are provided in the Zenodo repository in the directory Zenodo/ABFE_blobs/blobs_FF, where the ORAC prm/tpg and the GROMACS itp

files can be found.

Table S1: LJ and electrostatic blob parameters. The blobs are characterized by two atom types (q, n) with distinct LJ parameters and charges. B_{41}^{-1} has the same chemical topology as ligand 29 (see Figure 3 of the main paper) with $-0.5\ e$ on the oxygen atoms and zero charges on all others. For bonded parameters see the directory Zenodo/ABFE_blobs/blobs_FF in the Zenodo repository

	q^+	q^-	q_n	σ_q	σ_n	ϵ_q	ϵ_n	q_{tot}
B_{41}^{-1}	0	-0.5	0	[29]	[29]	[29]	[29]	-1
B_6^{-1}	0.8	-0.75	-0.1	3.0	3.6	0.15	0.05	-1
$B_6^{-0.5}$	0.7	-0.75	0.1	3.0	3.6	0.15	0.05	-0.5
B_1^0	n/a	n/a	0	n/a	3.92	n/a	0.5	0

The initial PDB structures of the bound states were taken from the GitHub site indicated in Ref.[3] for ABCG2 evaluation on the jacs-set.[4] The simulation boxes for bound and unbound states were cubic, with size chosen such that the distance from any solute atom (the complex or the ligand) to the box boundary did not exceed 12 Å, resulting in approximately 4300 and 900 water molecules for the bound and unbound states, respectively. Electrostatic interactions were treated with the particle mesh Ewald method using a grid size of $\simeq 1$ Å and a fourth-order B-spline for the gridded charge distributions. We used an r-RESPA multiple time-step integrator[5] specifically tuned for biological systems.[6] The system underwent a preliminary equilibration of the density in the NPT ensemble at 300 K using an isotropic Parrinello-Rahman Lagrangian[6] and a series of Nosé thermostats until stationarity of the running average of the box side length was achieved (typically 50-100 ps).

1.2 HREM simulations

Bound arm: HREM simulations of the bound state for the 16 ligands were performed with a solute tempering scaling protocol[7] using a minimum scaling factor of 0.15 for the torsional potential (corresponding to a maximum torsional "temperature" of 2000 K) and 0.33 for the intra-solute nonbonded interactions (corresponding to a maximum intra-solute nonbonded "temperature" of 1000 K). The "hot region" includes all residues in contact with the ligand in the starting NPT-equilibrated structure. A ligand-residue contact is assumed if

the distance from any atom of the ligand to any atom of the residue is less than 5.0 Å. Each run engaged 6 batteries of 16 replicates running for 5 ns, thus collecting 30 ns on the target state (T = 300 K, p = 1 atm) and approximately 0.5 μs in total across all replicas. In the scheme below we report the REM ORAC input for the bound state with the full definition of the hot region

```
&REM
BATTERIES  6
#  YOU WILL NEED NO LESS THAN 14 REPLICAS FOR THIS RUN
#  THE TOTAL NUMBER OF MPI PROCESSES SHOULD BE 140
#  THE TOTAL NUMBER OF CPU cores SHOULD BE 840
#  HREM SCALING BELOW
   SETUP    1.0           0.15         0.33        1
#  EXCHANGE FREQUENCY BELOW
   STEP  300.0
   PRINT_DIAGNOSTIC 1500.
#  THE HOT REGION BELOW
   SEGMENT
      define     1    41    ! lig    0
      define   933   936    ! ALA   58
      define   943   956    ! PHE   59
      define   987   997    ! MET   62
      define  1069  1081    ! LEU   66
      define  1243  1255    ! LEU   77
      define  1297  1306    ! VAL   80
      define  1313  1323    ! MET   81
      define  1366  1375    ! VAL   84
      define  1382  1395    ! PHE   85
```

```
    define  1507  1524   ! ARG   94
    define  1566  1573   ! THR   97
    define  1580  1592   ! LEU   98
    define  1629  1642   ! PHE  101
    define  1996  2008   ! ILE  125
    kind intra   # scaling only within the hot region
  END
  PRINT 1500.00
  PRINT_ENERGY 1500.0 OPEN 1.rem # print unscaled energy for MBAR
&END
```

The HREM hybrid OpenMP/MPI jobs were distributed over 768 cores (96 MPI tasks × 8 OpenMP threads) of the CRESCO8 cluster provided by ENEA[8] and were completed in 12-15 wall-clock hours. While the same protocol applies to B_{41}^{-1}, sampling of the simpler blobs (B_6^{-1}, $B_6^{-0.5}$, B_1^0) was achieved by running 16 conventional MD replicates in the NPT ensemble for 1 ns, thus collecting 16 ns in total and engaging 96 cores with a wall-clock time of 3-4 hours. In all equilibrium runs, the centers of mass (COMs) of the ligand (or blob) and the protein are connected via a flat-bottom potential with an allowance volume of approximately 800 Å^3, roughly corresponding to the MCL-1 binding site volume. The HREM sampling of the real ligands is the most demanding computational step of the entire DT-NE pipeline for recovering RBFEs. As stated in the theory section, the accuracy of the calculation depends crucially on this sampling.

Unbound arm: The scaling protocol for the solvated ligands and B_{41}^{-1} is the same as described above, with the hot region including the ligand only. Sixteen batteries of HREM simulations (8 replicas each) were run for 1 ns, collecting 16 ns on the target state. For B_6^{-1}, B_6^{-0-5}, and B_1^0, we ran 16 replicates of standard MD for 0.5 ns, for a total of 8 ns. The unbound state jobs required at most a few tens of wall-clock minutes for each ligand.

1.3 Preparation of DT starting configurations from HREM

The process for setting up the starting configurations for the NE-DT alchemy with a blob is schematically depicted in Fig. S1.

Bound arm: Let us denote the starting DT configurations of a tethered pair A-B as (A)-B and A-(B), where parentheses indicate the ghost (decoupled) partner. The starting DT configuration (blob)-L_i for the NE runs in the bound state (here, lowercase "blob" refers to any of the four blobs shown in Figure 3 of the main paper were prepared by combining 96 configurations sampled at regular intervals from the 30 ns HREM bound state simulation of the fully coupled ligand L_i with 96 configurations sampled from a 300 K gas-phase simulation of a single blob at T=300 K.

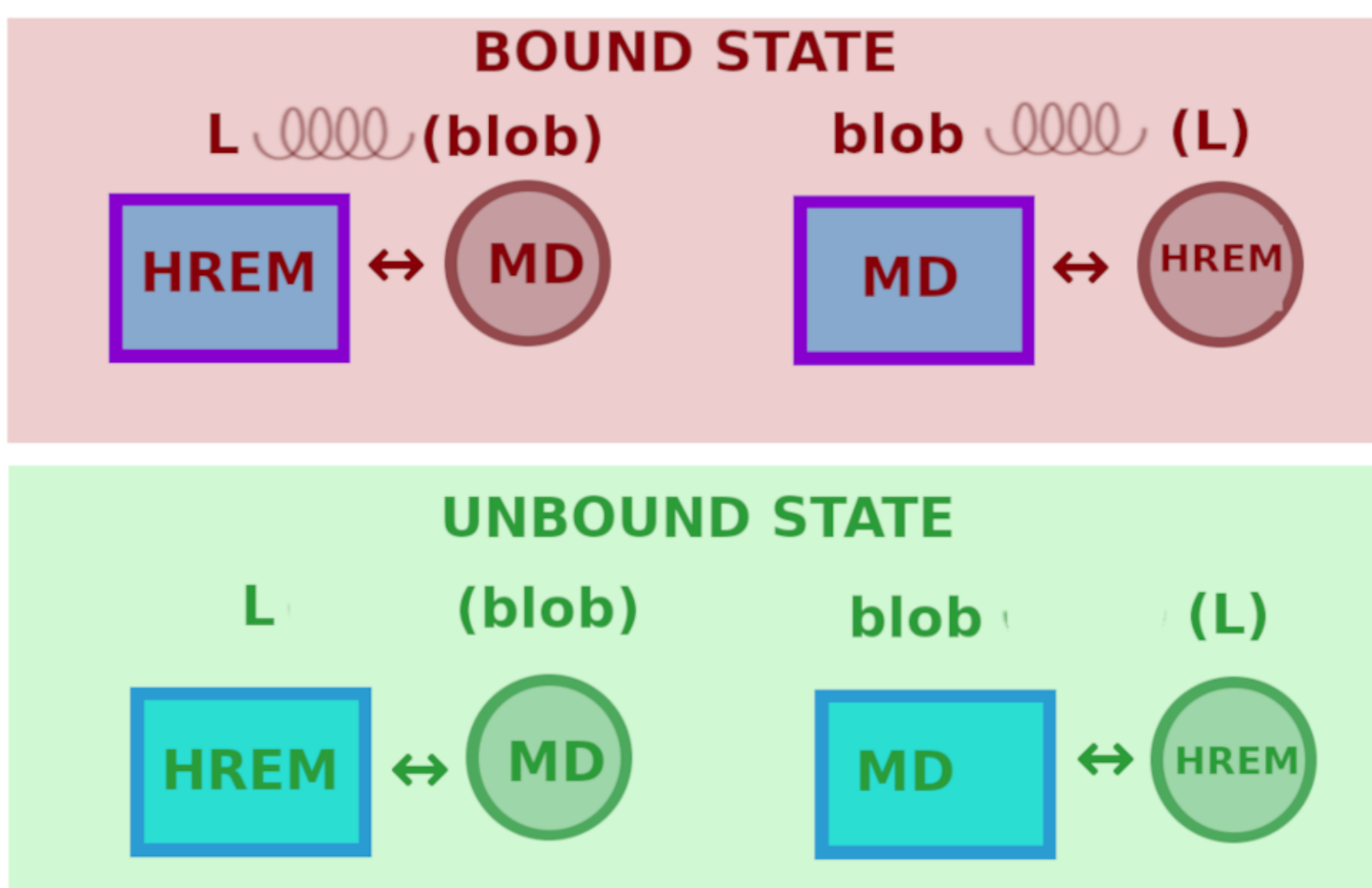

Figure S1: Starting configurations for the L(blob) and blob(L) DT partners in the bound and unbound arms of Figure 2 of the main paper. Rectangles and circles refer to complex and gas-phase configurations, respectively. Text in rectangles and circles indicates the type of sampling. Connected partners are marked by a spring symbol.

The COMs of the two partners are made to coincide, and a tethering COM-COM harmonic potential (see main paper) with $K = 2$ kcal/mol Å^{-2} is imposed. Similarly, the starting DT configuration blob-(L_i) in the bound state was constructed by combining 96

configurations of the fully coupled blob in the bound state with an equal number of gas-phase configurations of the ghost ligand L_i, connecting their coincident COMs by the harmonic potential. The ghost ligand L_i configurations are sampled from an 8-replica HREM 1 ns simulation of the isolated molecule at 300 K.

Unbound arm: Ninety-six starting configurations are prepared by adding the ghost partner (sampled from gas-phase simulations) at a random position to the configurations of the fully coupled ligand (sampled with HREM for real ligands). No tethering potential is imposed in this case.

1.4 DT-NE alchemical simulations and RBFE calculations

From the 96 DT configurations (blob)-L_i and blob-(L_i) in the bound state, we launched in parallel 96 NE alchemical transmutations, each lasting 0.72 ns. Electrostatic and LJ interactions of the ghost and fully coupled partners with the environment are turned off and on simultaneously, using a linear time protocol and the shifted-potential regularization (see main paper) to avoid singularities and reduce dissipation during the alchemical switch. Each OpenMP/MPI hybrid job engaged 768 cores on the CRESCO8 cluster and was completed in less than 2 wall-clock hours. The time record of the accumulated work on the driven λ parameter was recorded every 300 fs. A typical example of the work swarms for the $L_{29} \rightarrow$ BLOB transitions in bound and unbound state is shown in Figure S2.

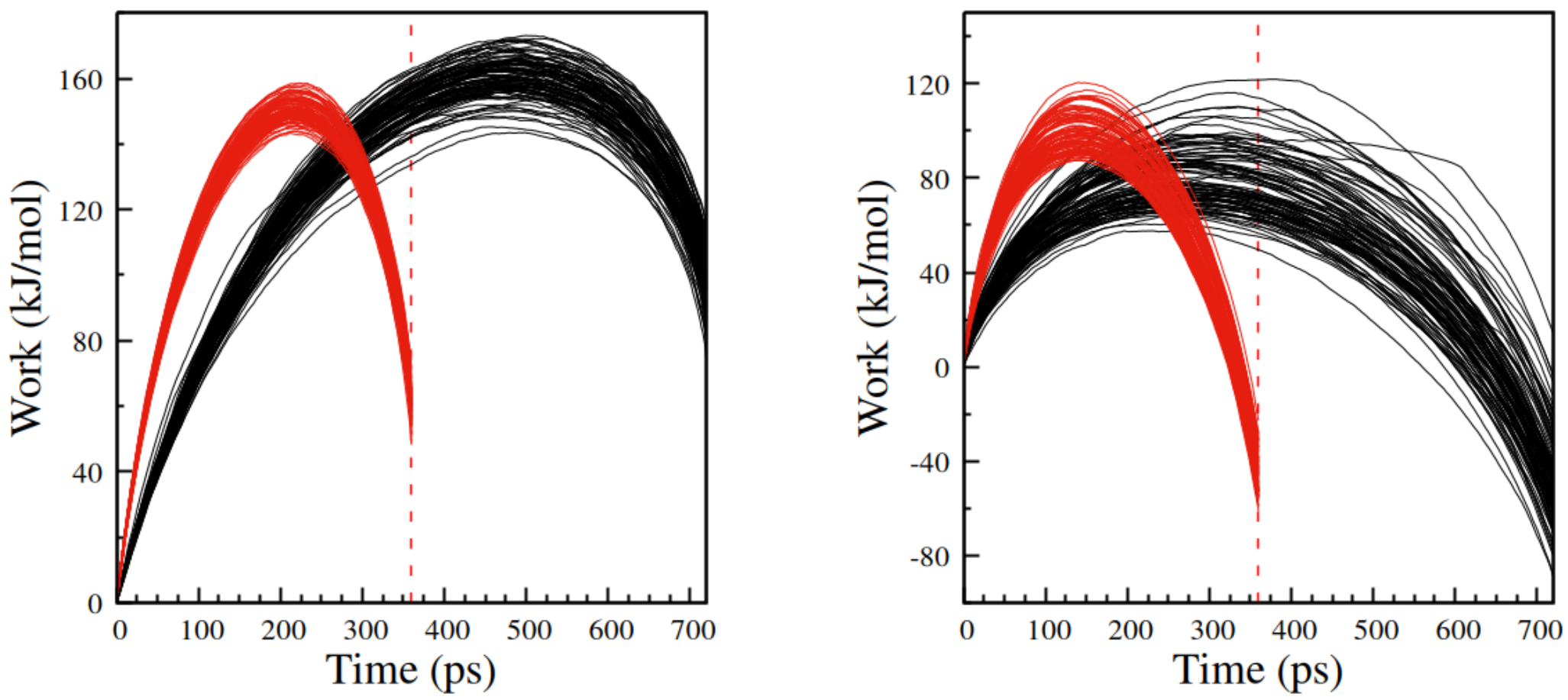

Figure S2: Time record of the work in the bound (black trait) and unbound (red trait) states during the alchemical transformation $L_{29} \rightarrow \mathrm{B}_6^{-1}$ (left) and $\mathrm{B}_6^{-1} \rightarrow L_{29}$ (right)

For the unbound state, we launched 96 blob $\rightarrow L_i$ and $L_i \rightarrow$ blob NE alchemical transmutations, each lasting 0.36 ns, with a linear switch protocol and the shifted potential regularization. In the example of Figure S2(b), we report the time record of the work swarm for the $L_{29} \rightarrow$ blob transition in the unbound state. Note that the dissipation (i.e., the spread of the work values at the end of the transition) is much higher in the bound state, despite the fact that the duration of the bound state NE transition is twice that of the unbound NE switch.

Once the NE runs were completed, four work samples were collected for each (blob, L_i) pair, corresponding to the forward and reverse processes $L_i \rightarrow$ blob and blob $\rightarrow L_i$ in the bound and unbound states. Prior to BAR calculations, the work collections were purged of potential outliers. Because the orientation and conformation of the ghost partner are initially random, an NE run may occasionally experience steric stresses that cannot be efficiently relaxed during the transition, leading to large positive work. Outliers were rare, and even when they did occur, they never compromised job completion. Work outliers can be detected with higher probability in the bound state, where steric clashes occurring during the re coupling process are less easily tamed due to the rigidity of the binding pocket.

The BAR calculation is performed on *two*, rather than four, work distributions. These are obtained by combining the bound work $W^{(b)}_{L\rightarrow \text{blob}}$ and unbound work $W^{(u)}_{\text{blob}\rightarrow L}$ values as $W = W^{(b)}_{L\rightarrow \text{blob}} + W^{(u)}_{\text{blob}\rightarrow L}$, and a reverse distribution with the labels A and B inverted everywhere. The resulting N^2 convolution histograms (see main paper) are related to the process of turning A into B in the bound state and B into A in the unbound state, or vice versa, as if these processes were *virtually* occurring in the same box. The corresponding BAR free energies are the RBFE and minus the RBFE between the L and blob pair.

The benefits of this technique compared to the standard "double system, single box" approach (implemented, e.g., in the `pmx` GROMACS ancillary tool for RBFE[9]), where the unbound A and B pair is explicitly present in a large box also containing the A-B complex, are threefold: (i) the BAR estimates are enhanced by the convolution, as the resulting work sample now includes N^2 instead of N values, thus increasing precision; (ii) the time protocols of the bound and unbound alchemical switches need not be identical, so that the less dissipative unbound process can be performed more rapidly; (iii) the box size can be individually adapted to the size of the solvated complex and the ligand in bulk, yielding a clear gain in computational efficiency compared to the single large box required to accommodate both systems in the pmx/GROMACS protocol.

2 Calculation of the ABFE of B_6^{-1}

ABFE of the B_6^{-1} is computed from a 288 work sample using the vDSSB approach.[10] Briefly, starting from the end-state configurations, the blob is decoupled in the bound state in 0.720 ns affording the bound state wok distribution $P_b(W)$. During the 288 NE simulations, the ligand is kept in the MCL-1 binding site by way a weak harmonic restraint with a force constant of 0.05 kcal/mol/Å^2 between the centers of mass (COM) of the ligand and the receptor. The re-coupling work distribution of the ligand in bulk solvent, $P_u(W)$, is obtained using 96 fast growth (0.36 ns) alchemical simulations. The starting configurations of the

decoupled blob in the unbound state are obtained by combining equilibrium configurations of TIP3P water (512 molecules) in the NPT ensemble with the gas-phase configurations of a single blob. The standard dissociation free energies, ΔG_0, are estimated using the Jarzynski estimate,[11] evaluated on the convolution $P_u(W) \star P_b(W)$ featuring a sample of 27648 vDSSB work values. In the Zenodo repository, we provide the script `vdssb.bash` to compute the ABFE of B_6^{-1} (as well as that of all the other listed blobs) from the raw work data. For more information on the repository structure, see Section S5.

3 Dissipation in the NE-DT transition with blobs

Dissipation in the DT-NE alchemical process is related to the variance σ^2 of the work distributions. The higher the variance, the further the mean work $\langle W \rangle$ is from the underlying free energy. In Figure S3, we report the spread (σ) of the work distributions for all blob $\rightarrow L_i$ and $L_i \rightarrow$ blob transmutations in both legs of the thermodynamic cycle of Figure 2 in the main paper.

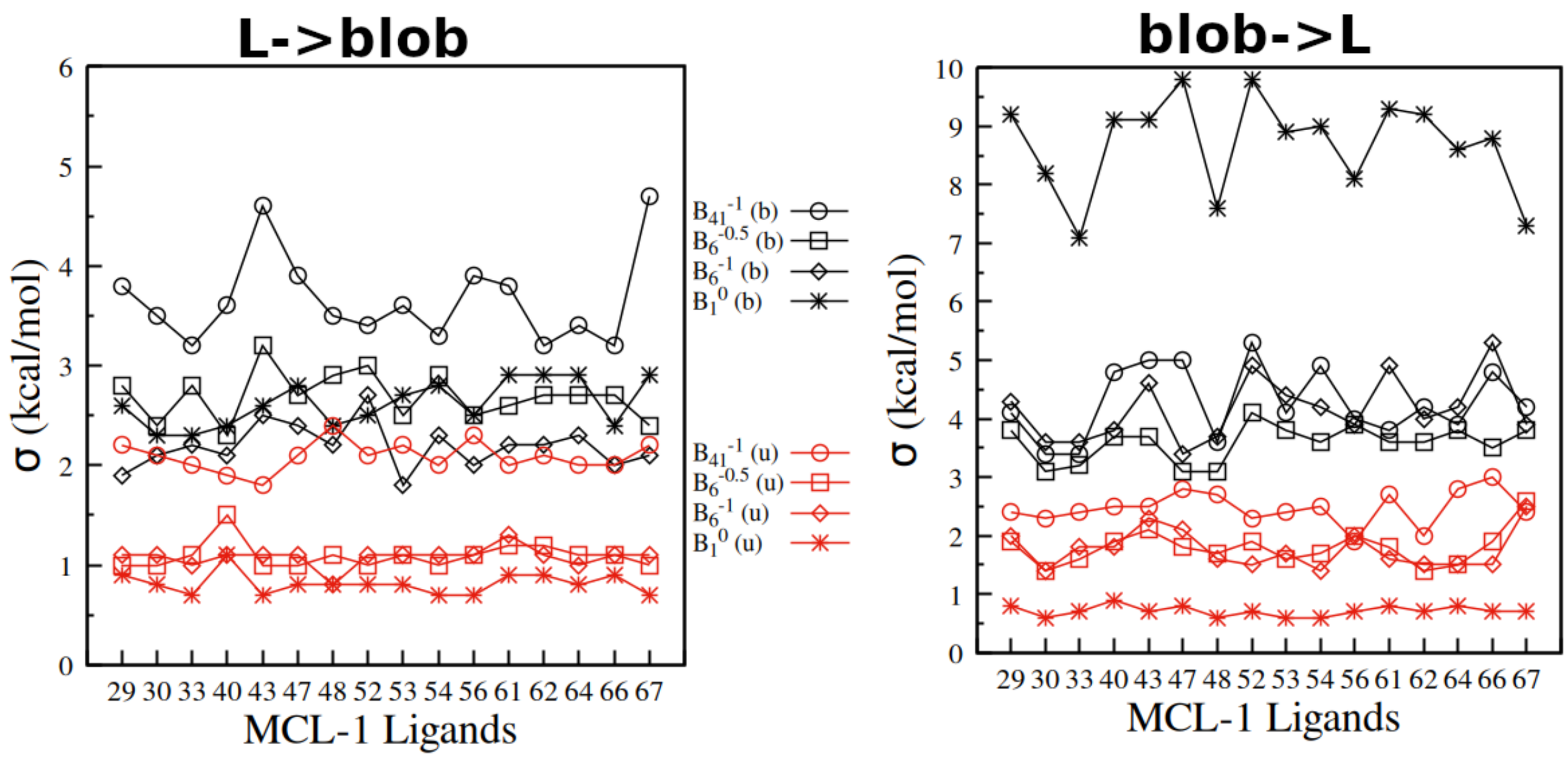

Figure S3: Spread (σ) in kcal/mol for the $L_i \to$ blob and blob $\to L_i$ NE processes in the bound (black trace) and unbound (red trace) states for the 16 ligands of the MCL-1 protein. The numbers on the x-axis refer to the ligands reported in Figure 3 of the main paper. The structures of the four blobs (B_n^q) are also shown in Figure 3 of the main paper. The data refer to the ABCG2 model.

4 AM1BCC/ABCG2 correlation for the two-edge RBFEs of ligands in Figure 3 of the main paper

In Figure S4, we report the correlation and related metrics for the 120 RBFEs involving the ligands shown in Figure 3 of the main paper, calculated using AM1BCC and ABCG2 bond charge corrections for the two small blobs B_1^0 and B_6^{-1}. The data are strongly linearly related, and most of the differences between the two models lie within the corresponding BAR-based confidence intervals. This is especially true for the simple neutral LJ blob B_1^0, which exhibits large dissipation in the $B_1^0 \to L_i$ transition (see Figure S3).

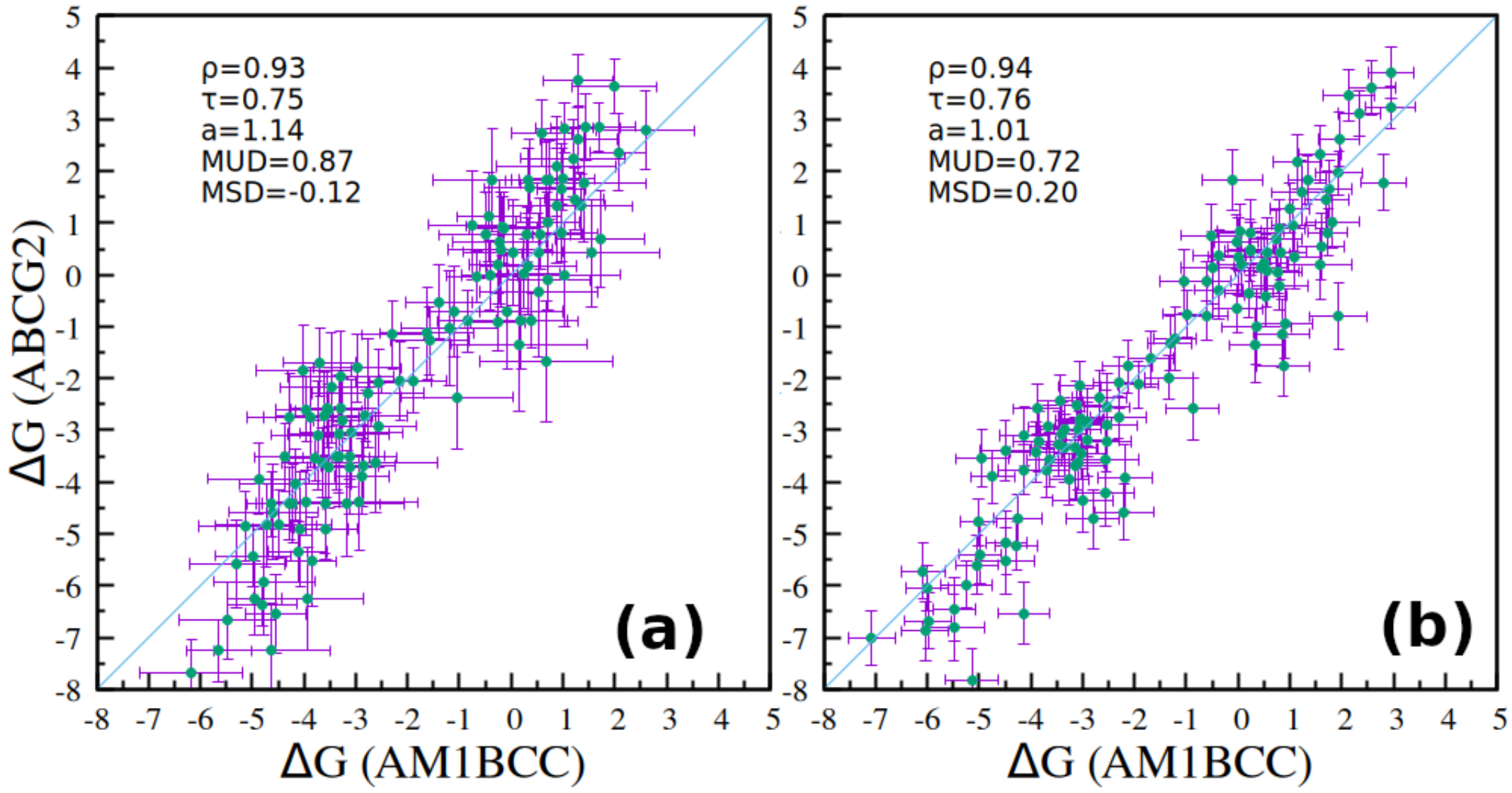

Figure S4: AM1BCC vs. ABCG2 correlation plot for RBFEs using B_1^0 (a) and B_6^{-1} (b). ΔG, MUD, and MSE are in units of kcal/mol.

5 Directory tree of the Zenodo repository

In the following we show the directory tree of the Zenodo repository. Each directory contains a `README` file providing further for information. Results reported in the paper can be reproduced from the raw work data using the s/w provided in the `bin` of the Zenodo repository

```
Zenodo
|— ABFE_blobs    contains all data for ABFE calculations
|     |— bl6q  *
|     |— blb1
|     |— blb2  *
|     |— blb6  *
|     |— bllj
|     |— blob
```

```
│   ├── ljbl  *
│   ├── blobs_FF   contains the blobs' FF parameters
│   ├── data_abs
│   │   ├── abcg2
│   │   │   └── TABLES
│   │   └── am1bcc
│   │       └── TABLES
│   ├── Datap           contains the raw work data for ABFE
├── bin        contains ancillary s/w to reproduce the results
│   ├── BAR
│   │   └── sample-data
│   ├── EM
│   │   ├── sample-data
│   │   └── src
│   └── QQ_test
├── doc
├── pdb
├── RBFE    contains all data for RBFE calculations
│   ├── ABCG2
│   │   ├── data
│   │   │   └── TABLES
│   │   ├── Datarel contains the raw work data for RBFE
│   │   ├── lib  contains the ligands' FF parameters
│   │   └── vdssb
│   ├── AM1BCC
│   │   ├── data
│   │   │   └── TABLES
```

```
│    │    │
│    │    ├── Datarel  contains the raw work data for RBFE
│    │    ├── lib  contains the ligands' FF parameters
│    │    └── vdssb
│    ├── gplt
│    ├── input_examples  contains ORAC input examples
│    │    ├── HREM
│    │    └── NE
│    │         ├── b-pdbs
│    │         └── u-pdbs
│    └── NE-paths
├── ST_data   contains Behera et al. [Ref 3] data for ST-RBFE
└── zip   contains the orac program
```

References

(1) GAFF and GAFF2 are public domain force fields and are part of the AmberTools distribution, available for download at https://amber.org internet address (accessed January, 2022). According to the AMBER development team, the improved version of GAFF, GAFF2, is an ongoing poject aimed at "reproducing both the high quality interaction energies and key liquid properties such as density, heat of vaporization and hydration free energy". GAFF2 is expected "to be an even more successful general purpose force field and that GAFF2-based scoring functions will significantly improve the successful rate of virtual screenings.".

(2) Lindorff-Larsen, K.; Piana, S.; Palmo, K.; Maragakis, P.; Klepeis, J. L.; Dror, R. O.; Shaw, D. E. Improved side-chain torsion potentials for the Amber ff99SB protein force field. *Proteins* **2010**, *78*, 1950–1958.

(3) Behera, S.; Gapsys, V.; de Groot, B. L. Evaluation of the ABCG2 Charge Model in

Protein–Ligand Binding Free-Energy Calculations. *J. Chem. Inf. and Model.* **2025**, *65*, 11505–11509.

(4) Wang, L.; Wu, Y.; Deng, Y.; Kim, B.; Pierce, L.; Krilov, G.; Lupyan, D.; Robinson, S.; Dahlgren, M. K.; Greenwood, J.; Romero, D. L.; Masse, C.; Knight, J. L.; Steinbrecher, T.; Beuming, T.; Damm, W.; Harder, E.; Sherman, W.; Brewer, M.; Wester, R.; Murcko, M.; Frye, L.; Farid, R.; Lin, T.; Mobley, D. L.; Jorgensen, W. L.; Berne, B. J.; Friesner, R. A.; Abel, R. Accurate and Reliable Prediction of Relative Ligand Binding Potency in Prospective Drug Discovery by Way of a Modern Free-Energy Calculation Protocol and Force Field. *J. Am. Chem. Soc.* **2015**, *137*, 2695–2703.

(5) Tuckerman, M.; Berne, B. J. Reversible multiple time scale molecular dynamics. *J. Chem. Phys.* **1992**, *97*, 1990–2001.

(6) Marchi, M.; Procacci, P. Coordinates scaling and multiple time step algorithms for simulation of solvated proteins in the NPT ensemble. *J. Chem. Phys.* **1998**, *109*, 5194–520g2.

(7) Marsili, S.; Signorini, G. F.; Chelli, R.; Marchi, M.; Procacci, P. ORAC: A Molecular Dynamics Simulation Program to Explore Free Energy Surfaces in Biomolecular Systems at the Atomistic Level. *J. Comput. Chem.* **2010**, *31*, 1106–1116.

(8) Iannone, F.; Ambrosino, F.; Bracco, G.; De Rosa, M.; Funel, A.; Guarnieri, G.; Migliori, S.; Palombi, F.; Ponti, G.; Santomauro, G.; Procacci, P. CRESCO ENEA HPC clusters: a working example of a multifabric GPFS Spectrum Scale layout. 2019 International Conference on High Performance Computing Simulation (HPCS). 2019; pp 1051–1052.

(9) Gapsys, V.; Michielssens, S.; Seeliger, D.; de Groot, B. L. pmx: Automated protein structure and topology generation for alchemical perturbations. *J. Comp. Chem.* **2015**, *36*, 348–354.

(10) Macchiagodena, M.; Pagliai, M.; Karrenbrock, M.; Guarnieri, G.; Iannone, F.; Procacci, P. Virtual Double-System Single-Box: A Nonequilibrium Alchemical Technique for Absolute Binding Free Energy Calculations: Application to Ligands of the SARS-CoV-2 Main Protease. *J. Chem. Theory Comput.* **2020**, *16*, 7160–7172.

(11) Jarzynski, C. Nonequilibrium equality for Free energy differences. *Phys. Rev. Lett.* **1997**, *78*, 2690–2693.
